# Amnion-Epithelial-Cell-Derived Exosomes Demonstrate Physiologic State of Cell under Oxidative Stress

**DOI:** 10.1371/journal.pone.0157614

**Published:** 2016-06-22

**Authors:** Samantha Sheller, John Papaconstantinou, Rheanna Urrabaz-Garza, Lauren Richardson, George Saade, Carlos Salomon, Ramkumar Menon

**Affiliations:** 1 Division of Maternal-Fetal Medicine & Perinatal Research, Department of Obstetrics & Gynecology, The University of Texas Medical Branch at Galveston, Galveston, Texas, United States of America; 2 Department of Biochemistry and Molecular Biology, The University of Texas Medical Branch at Galveston, Galveston, Texas, United States of America; 3 Exosome Biology Laboratory, Centre for Clinical Diagnostics, UQ Centre for Clinical Research, Faculty of Health Sciences, University of Queensland, Herston, Queensland, Australia; Shanghai Jiaotong University School of Medicine, CHINA

## Abstract

At term, the signals of fetal maturity and feto-placental tissue aging prompt uterine readiness for delivery by transitioning quiescent myometrium to an active stage. It is still unclear how the signals reach the distant myometrium. Exosomes are a specific type of extracellular vesicle (EVs) that transport molecular signals between cells, and are released from a wide range of cells, including the maternal and fetal cells. In this study, we hypothesize that *i*) exosomes act as carriers of signals in utero-placental compartments and *ii*) exosomes reflect the physiologic status of the origin cells. The primary aims of this study were to determine exosomal contents in exosomes derived from primary amnion epithelial cells (AEC). We also determined the effect of oxidative stress on AEC derived exosomal cargo contents. AEC were isolated from amniotic membrane obtained from normal, term, not in labor placentae at delivery, and culture under standard conditions. Oxidative stress was induced using cigarette smoke extract for 48 hours. AEC-conditioned media were collected and exosomes isolated by differential centrifugations. Both growth conditions (normal and oxidative stress induced) produced cup shaped exosomes of around 50 nm, expressed exosomes enriched markers, such as CD9, CD63, CD81 and HSC70, embryonic stem cell marker Nanog, and contained similar amounts of cell free AEC DNA. Using confocal microscopy, the colocalization of histone (H) 3, heat shock protein (HSP) 70 and activated form of pro-senescence and term parturition associated marker p38 mitogen activated protein kinase (MAPK) (P-p38 MAPK) co-localized with exosome enrich marker CD9. HSP70 and P-p38 MAPK were significantly higher in exosomes from AEC grown under oxidative stress conditions than standard conditions (p<0.05). Finally, mass spectrometry and bioinformatics analysis identified 221 different proteins involved in immunomodulatory response and cell-to-cell communication. This study determined AEC exosome characteristics and their cargo reflected the physiologic status of the cell of origin and suggests that AEC-derived exosomal p38 MAPK plays a major role in determining the fate of pregnancy. Understanding the propagation of fetal signals and their mechanisms in normal term pregnancies can provide insights into pathologic activation of such signals associated with spontaneous preterm parturitions.

## Introduction

Normal term human parturition is initiated between 37 and 40 weeks of gestation when in utero fetal growth and development are completed [1–3]. The signals from mature fetal organs prompt uterine readiness for delivery by transitioning quiescent myometrium to an active stage (contractile phenotype) [2,4,5]. Various endocrine, immune and mechanical signals from feto-maternal compartments enhance overall uterine inflammatory load leading to functional progesterone withdrawal causing myometrial contractility [6–8]. Therefore, term labor and delivery results from well-orchestrated activities of various endocrine and paracrine factors. Nonetheless, the signature of these signals and their precise mechanisms in initiating parturition are still unclear and under investigation by many laboratories. Understanding the mechanisms of these signals in normal term pregnancies can provide insights into pathologic activation that can cause spontaneous preterm parturitions.

Recently, our laboratory has reported fetal membrane (amniochorionic membrane) senescence as a mechanism associated with normal term parturition [9,10]. Placental membranes undergo an oxidative stress associated telomere dependent cellular senescence at term [11–13]. In addition, placental membrane senescence is also associated with sterile inflammation in the amniotic fluid [9,14]The unique inflammation seen in senescent cells is defined senescence associated secretory phenotype (SASP) [15]. SASP is characterized by proinflammatory cytokines and chemokines that are reported to be associated with term labor and delivery [9]. The findings in clinical specimens have been reproduced in vitro in primary amnion epithelial cells in culture exposed to oxidative stress induced by cigarette smoke extract (CSE) [12]. Generation of senescence and SASP were reduced by inhibiting p38 mitogen activated kinase (MAPK), a stress associated pro-senescence protein, suggesting that sterile inflammation can be generated by senescent cells [12,14,16].

It is still unclear how proinflammatory SASP signals from senescent fetal membranes can reach distant myometrium or whether they are confined to enforcing local (fetal) tissue damage and inflammation until parturition. Senescent signals may be transported to distant tissues indicating a dysfunctional fetal membrane status that prompts delivery of the fetus, as well as placenta and membrane. Although localized activities of SASP can be achieved through direct cell-cell contact or through ligand-receptor interactions, distant feto-maternal communication is likely facilitated through specific carriers that can transport and deliver signals from senescent cells. Thus, prior to projecting senescent fetal cells signaling parturition at a distant myometrium, the mode of delivery of such signals must be established. Several recent reports and reviews propose a role for exosomes as such carriers of parturition signals to utero-placental compartments [17–20].

Exosomes are 30–100 nm endosome-derived vesicles with specific characteristics that separate them from other larger particles such as microvesicles and apoptotic bodies [21–24]. Exosome biogenesis is a process that begins with the endocytosis of transmembrane proteins [25,26]. Once sorted to late endosomes, the endosomal sorting complex required for transport (ESCRT) complex, recruits proteins and other cargo, while also mediating the inward budding of the late endosome, creating the intraluminal vesicles inside the multivesicular body (MVB) [27–30]. The MVB can either follow a degradation pathway fusing with lysosomes or proceed to release the intraluminal vesicles into the extracellular space through exocytosis as exosomes [27,28]. Placental derived exosomes have been well characterized during normal and abnormal pregnancies and their functional roles have also been documented [4–13,31–39]. Their size facilitates easy transportation between cells and tissues, while their contents reflect the state of the source cell and regulate the phenotype of the target cell [23,34,36,40–42]. Exosomes interact with the target cell by direct fusion with the cell membrane, thus releasing the contents directly into the cytosol; through active uptake via endocytosis or by binding to the target cell via receptor-ligand interactions thereby inducing a signaling cascade which changes the phenotype of the target cell.

No reports exist on fetal membrane- derived exosomes or their contents. Therefore, the objectives of this study are: 1) determine the generation of exosomes from primary amnion epithelial cells and characterize their contents, 2) determine the changes in specific exosome contents in primary amnion cells in response to oxidative stress. Since p38 MAPK was identified as a crucial stress response-signaling pathway that activates oxidative stress induced senescence at term, we specifically examined exosomal p38 MAPK cargo. Using an in vitro primary amnion epithelial cell (AEC) model of oxidative stress induced by cigarette smoke extract, a well characterized model of in vitro oxidative stress, and using Liquid Chromatography (LC)/ Mass Spectrometry (MS) we characterize the contents of AEC -derived exosomes and their potential role in parturition.

## Materials and Methods

Placental samples were obtained for this study from John Sealy Hospital at The University of Texas Medical Branch (UTMB) at Galveston, TX, USA. No subjects were recruited or consented for this study as we used discarded placenta from normal term, not in labor cesarean sections. The study protocol was submitted and approved by the institutional review board at UTMB, whereby placental samples could be collected without consenting subjects. Placentae from women (18–40 years old) undergoing elective repeat cesarean delivery (between 37 and 41 weeks of gestation) prior to the onset of labor were included in the study. Women with prior history of preterm labor and delivery, preterm premature rupture of the membranes, preeclampsia, placental abruption, intrauterine growth restriction, and gestational diabetes were excluded. Additionally, group B *streptococcus* carriers, treated for urinary tract infection, sexually transmitted diseases, chronic infections like HIV, hepatitis, and women who smoked cigarettes or reported drug and alcohol abuse, were also excluded.

### Isolation and Culture of human Amnion Epithelial Cells (AECs)

All reagents and media were warmed to 37°C prior to use. The amniotic membrane was processed as described previously [6, 12, 14] Briefly, the amnion membrane was manually peeled from normal, term, not in labor caesarean section placentas, rinsed in saline and transferred to a petri dish containing Hanks Balanced Salt Solution (HBSS; Mediatech Inc., Manassas, VA). After cutting the amnion into 2 cm x 2 cm pieces, they were digested twice in 0.25% trypsin and 0.125% Collagenase A (Sigma–Aldrich, St. Louis, MO) in HBSS for 35 minutes at 37°C. After each digestion, the tissue was filtered through a 70 μm cell strainer (Thermo Fisher Scientific, Waltham, MA) and trypsin was inactivated using complete Dulbecco's Modified Eagle Medium: Nutrient Mixture F-12 media (DMEM/F12; Mediatech Inc.) supplemented with 15% fetal bovine serum (FBS; Sigma-Aldrich), 10% Penicillin/Streptomycin (Mediatech Inc.) and 100 μg/mL epidermal growth factor (EGF; Sigma-Aldrich). The collected filtrate was centrifuged for 10 minutes at 3000 RPM and the pellet was resuspended in 3.0 mL complete DMEM/F12. Once cells were counted, approximately 3–5 million cells per flask were cultured in T75 flasks containing complete DMEM/F12 media at 37°C, 5% CO_2_, and 95% air humidity to 70–80% confluence.

To ensure the purity of our primary AEC cultures, immunofluorescent staining was performed. Cells were seeded on glass coverslips at a density of 30,000 cells per slip and incubated overnight. Cells were fixed with 4% paraformaldehyde (PFA), permeablized with 0.5% Triton X and blocked with 3% BSA in PBS prior to incubation with Cytokeratin 18 (Abcam, Cambridge, United Kingdom) primary antibody diluted 1:300 in 3% BSA overnight at 4°C. After washing with PBS, slides were incubated Alexa Fluor conjugated secondary antibodies (Life Technologies, Carlsbad, CA) diluted 1:400 in PBS for 1 hour in the dark. Slides were washed with PBS then treated with NucBlue® Live ReadyProbes® Reagent (Life Technologies) then mounted using Mowiol 4–88 mounting medium (Sigma-Aldrich). Images were captured using LSM 510 Meta UV confocal microscope (63x) (Zeiss, Germany).

### Stimulation of AEC with cigarette smoke extract (CSE)

To induce oxidative stress in AECs, CSE was used as detailed in our prior studies, [12,43,44] with modifications. Smoke from a single lit commercial cigarette (unfiltered Camel^TM^, R.J. Reynolds Tobacco Co, Winston Salem, NC) was infused into 25 mL of exosome-free media, consisting of DMEM/F12 supplemented with 10% exosome-free FBS (System Biosciences, Mountain View, CA). The stock CSE was sterilized using 0.25 mm Steriflip® filter unit (Millipore, Billerica, MA). CSE concentrate was diluted 1:10 in exosome-free media prior to use. Once cells reached 70–80% confluence, each flask was rinsed with sterile 1x PBS followed by treatment with exosome-free media (control) or CSE containing media and incubated at 37°C, 5% CO_2_, and 95% air humidity for 48 hours.

### Cell cycle analysis of AECs using flow cytometry

CSE treated and control AECs were harvested after media collection using trypsin EDTA (Corning, Corning, NY) and centrifuged for 10 minutes at 3000 RPM. The supernatant was removed and cells were resuspended in 50 μL PBS. Cell cycle analysis was performed using the Coulter DNA Prep Reagents Kit (Beckman Coulter, Indianapolis, IN). Briefly, 50 μL of DNA Prep LPR was added to each sample and vortexed. Then 1.0 mL DNA Prep Stain was added to the tubes, vortexed and run immediately on the Cytoflex flow cytometer (Beckman Coulter). After selecting for single cells, gating was set for the control cells and applied to histograms for the CSE treated AECs using Cytexpert (Beckman Coulter).

### Activation of p38 MAPK in AECs using flow cytometry

Activation of p38 MAPK was also performed. After harvesting cells using trypsin EDTA and centrifugation for 10 minutes at 3000 RPM, the pellet was resuspended in 500 μL 4% paraformaldehyde and vortexed. After incubation for 10 minutes at room temperature, cells were placed on ice for 1 minute then centrifuged for 5 minutes at 2000 RPM at 4°C. The supernatant was removed and the pellet was resuspended in 500 μL 90% ice cold methanol, gently vortexing while adding methanol slowly. Once the pellet was completely resuspended, the cells were incubated on ice for 10 minutes then stored at -20°C until use.

All centrifugations were performed at 2000 RPM for 5 minutes at 4°C. Cells in 90% methanol were centrifuged and washed twice with 5% BSA in PBS. After the second centrifugation, the pellet was resuspended in P-p38 MAPK primary antibody (Cell Signaling) diluted 1:200 in 5% BSA and incubated at room temperature for 2 hours. Cells were washed twice with 5% BSA in PBS then resuspended in Alexa Fluor conjugated secondary antibody (Life Technologies) diluted 1:400 in PBS and incubated for 1 hour at room temperature in the dark. Cells were centrifuged and resuspended in 400 μL PBS and run immediately on the Cytoflex flow cytometer (Beckman Coulter). After gating for single cells, data analysis based was performed using Cytexpert (Beckman Coulter). Flow Cytometry analysis for exosome markers and DNA

### Exosome isolation

The culture media were collected and stored at -80°C until exosome isolation. Media was thawed overnight then isolated using differential ultracentrifugation as described previously, with modifications [21,45,46]. Briefly, the culture media was centrifuged sequentially at 4°C for 10 minutes at 300g and 20 minutes at 2,000g using Sorvall Legend X1R and TX-400 swinging bucket rotor (Thermo Fisher Scientific), 30 minutes at 10,000g and 2 hours at 100,000g using a Beckman Optima LX-80 ultracentrifuge with 50.1Ti and 70.1Ti rotors (Beckman Coulter). The pellet was resuspended in 1x PBS then centrifuged again at 100,000g for 1 hour. Pellet was resuspended in RIPA (Western Blot) or 1x PBS (electron microscopy, Flow Cytometry, DNA quant and sizing).

### Transmission Electron Microscopy (TEM) of whole mounted exosomes

For TEM studies, 5 μL suspended exosomes in PBS were dropped onto a formvar-carbon coated 300-mesh copper grid and left to dry at room temperature for 10 min. Grids were treated with 10 seconds of Hydrogen-Oxygen plasma in a Gatan Solarus 950 plasma cleaning system (Gatan, Inc., Pleasanton, CA) prior to use. After three washes in purified water, the exosome samples were negatively stained using PhosphoTungstic Acid (PTA). The grids were dried at room temperature then viewed in a 120 keV JEM 1400 electron microscope (Jeol, Peabody, MA). A minimum of 10 frames were viewed per sample.

### Exosome particle sizing analysis

Dynamic light scattering analysis (DLS) was used to determine the mean size distribution and purity of our exosome preps. 50 μL of concentrated exosomes was brought to a volume of 1.0 mL in 1x PBS and sized using Malvern High Performance Particle Sizer (HPPS) (Malvern Instruments, Worcestershire, UK).

### Western blot analysis

Exosomal pellets were resuspended in RIPA lysis buffer (50 mM Tris pH 8.0, 150 mM NaCl, 1% Triton X-100, and 1.0 mM EDTA pH 8.0, 0.1% SDS) supplemented with protease and phosphatase inhibitor cocktail and PMSF. After centrifugation at 10,000 RPM for 20 minutes, the supernatant was collected and protein concentrations were determined using BCA (Pierce, Rockford, IL). The protein samples were separated using SDS-PAGE on a gradient (4–15%) Mini-PROTEAN® TGX™ Precast Gels (Bio-Rad, Hercules, CA) and transferred to the membrane using iBlot® Gel Transfer Device (Thermo Fisher Scientific). Membranes were blocked in 5% nonfat milk in 1x Tris buffered saline-Tween 20 (TBS-T) buffer for a minimum of 1 h at room temperature then probed (or re-probed) with primary antibody overnight at 4°C. The membrane was incubated with suitable secondary antibody conjugated with horseradish peroxidase and immunoreactive proteins were visualized using Luminata Forte Western HRP substrate (Millipore, Billerica, MA). The stripping protocol followed the instructions of Restore Western Blot Stripping Buffer (Thermo Fisher). No blots were used more than three times.

The following anti-human antibodies were used for western blot: Exosome markers CD81 (Abnova, Taiwan) and heat shock cognate (HSC) 70 (Abcam) were diluted 1:500, while ESCRT complex protein Alix (Santa Cruz Biotechnology, Dallas, TX) was diluted 1:200. Embryonic stem cell markers Nanog andOCT-4, (Cell Signaling, Beverly, MA) were diluted 1:500. Heat shock protein (HSP) 70 (Abcam), a damage associated molecular pattern, was diluted 1:500. Total p38 MAPK and phospho p38 MAPK (Cell Signaling) were diluted 1:400.

### Flow Cytometry analysis for exosome markers and DNA

For flow cytometry analysis of exosome tetraspanin markers CD9, CD63 and CD81, a total of five samples per condition were prepared using the ExoFlow kit (System Biosciences) protocol with modifications. Briefly, exosomes isolated from treated and untreated amnion cell cultures were resuspended in 150 μL 1x PBS and all kit reagent volumes were halved. Streptavidin coated 9.1μm beads were washed then incubated with biotinylated anti-CD9, CD63 or CD81 for 2 hours on ice, flicking intermittently to mix. Beads were washed and resuspended in 200 μL bead wash buffer prior to incubation overnight at 4°C with 50 μL exosomes (total volume 250 μL). The following day, samples were washed and stained using the Exo-FITC exosome stain according to manufacturer then run on the Cytoflex flow cytometer (Beckman Coulter). Negative controls with isotype-matched antibodies were used for gating, applied according to manufacturer instructions. Data analysis based on fluorescein isothiocyanate (FITC) signal shift was performed using Cytexpert (Beckman Coulter).

After initial analysis for tetraspanin markers, the samples were tested for DNA content using the Coulter DNA Prep Reagents Kit (Beckman Coulter), which uses propidium iodide to tag DNA. Since DNA fragments are expected to stick to exosomal membranes and can confound with true exosomal DNA cargo, AEC exosomes were pretreated with DNase to remove all external DNA. To address the potential contamination, the samples were split in half, one set of samples were pretreated with DNase while the other half remained untreated. Pretreated samples were washed with exosome wash buffer provided in the ExoFlow kit then resuspended in DNase 1 set (Zymo Research, Irvine, CA) solution according to the manufacturer’s protocol, substituting exosome wash buffer for ethanol. Samples were incubated at room temperature for 10 minutes. To inactivate the DNase, samples were heated to 65°C for 15 minutes. After washing with exosome wash buffer three times, the pretreated and untreated exosomes were resuspended in 50 μL PBS then stained using the Coulter DNA Prep kit (Beckman Coulter). Samples were run on the Beckman Coulter CytoFlex Flow Cytometer. Negative controls, consisting of beads without exosomes attached, were used for gating, applied according to ExoFlow (SBI) instructions.

### Quantitation of exosomal DNA

To quantify the amount of DNA, exosomes were resuspended in 200 μL 1x PBS. DNA was extracted following DNeasy Blood and Tissue kit protocol (Qiagen, Hilden, Germany). Quantification was performed on the Synergy H4 Hybrid Microplate Reader (Biotek, Winooski, VT) using the Take 3 microvolume plate and Gen5 software (Biotek). We corrected for the blank and DNA concentration was calculated based on the absorbance ratio of 280/260.

### Immunofluorescence staining and microscopy

Cells were seeded on glass coverslips at a density of 30,000 cells per slip and incubated overnight prior to treatment with either exosome-free media (control) or 1:10 dilution of fresh CSE in exosome-free media. After 24 hour treatment, cells were fixed with 4% paraformaldehyde (PFA), permeablized with 0.5% Triton X and blocked with 3% BSA in PBS prior to incubation with primary antibodies overnight at 4°C. After washing with PBS, slides were incubated Alexa Fluor 488– or 594–conjugated secondary antibodies (Life Technologies) diluted 1:400 in PBS for 1 hour in the dark. Slides were washed with PBS then treated with NucBlue® Live ReadyProbes® Reagent (Life Technologies) then mounted using Mowiol 4–88 mounting medium (Sigma-Aldrich). Images were captured using LSM 510 Meta UV confocal microscope (63x) (Zeiss, Germany). Exosomes were tagged using anti-CD9 diluted 1:300, while P-p38 MAPK, Histone 3 (H3), and HSP70 were diluted 1:250. Colocalization of proteins of interest with exosomes was determined using LSM software (Zeiss) and Image J (open source). A total of five images per condition and 5 regions of interest per image were used to determine red and green fluorescence intensity values per distance (μm). Intensity was graphed versus distance for both colors using GraphPad Prism (GraphPad Software, La Jolla, CA). Areas of overlap indicate colocalization. The images were also analyzed for Pearson's Correlation Coefficient using Coloc 2 from Fiji (open source), selecting 5 regions of interest for each image. Mean Pearson’s values were graphed using Excel. Any image modifications (brightness, contrast, and smoothing) were applied to the entire image using Image J (open source).

### Proteomic analysis of AEC-derived exosomes by mass spectrometry

Protein profile of exosomes isolated from AEC culture under normal or oxidative stress conditions were established by Liquid Chromatography (LC)/ Mass Spectrometry (MS) as previously described with modifications [41,42]. Briefly, exosome pellets were lysed in 500μL modified RIPA buffer (2.0% SDS, 150mM NaCl, 50mM Tris, pH 8.5, 1X Complete Protease inhibitor (Roche)) at 100°C for 15 minutes. The lysate was clarified by centrifugation and the protein concentration determined by Qubit fluorometry (Invitrogen). 10 μg of extracted protein was processed by SDS-PAGE using 10% Bis Tris NuPage mini-gel (Invitrogen) in the MES buffer system. The migration window (2cm lane) was excised and in-gel digestion performed using a ProGest robot (DigiLab) using ammonium bicarbonate (25mM), dithiothreitol (reduction step, 10mM at 60°C) and iodoacetamide (alkylation step, 50mM). Samples were digested with sequencing grade trypsin (Promega) at 37°C for 4h and quenched with formic acid. The supernatant was analyzed directly without further processing. Digested samples were analyzed by nano LC-MS/MS with a Waters NanoAcquity HPLC system interfaced to a ThermoFisher Q Exactive. Peptides were loaded on a trapping column and eluted over a 75μm analytical column at 350nL/min using a 2hr reverse phase gradient; both columns were packed with Jupiter Proteo resin (Phenomenex). The mass spectrometer was operated in data-dependent mode, with the Orbitrap operating at 60,000 FWHM and 17,500 FWHM for MS and MS/MS respectively. The fifteen most abundant ions were selected for MS/MS. False discovery rate (FDR) was estimated using a reversed sequence database.

### Functional analysis of exosome proteome

Proteins identified by MS/MS were analyzed by PANTHER (Protein Analysis THrough Evolutionary Relationships; http://www.pantherdb.org) as previously described [41,42]. Differentially identified proteins were analyzed further by bioinformatic pathway analysis (Ingenuity Pathway Analysis [IPA]; Ingenuity Systems, Mountain View, CA; www.ingenuity.com).

### Statistical analysis

SPSS software (IBM, Armonk, NY) was used for statistical evaluation. Samples were analyzed using paired *t*–test and a *P* value less than 0.05 was considered statistically significant.

## Results

### Primary AEC cultures are positive for cytokeratin

**Fig 1:**
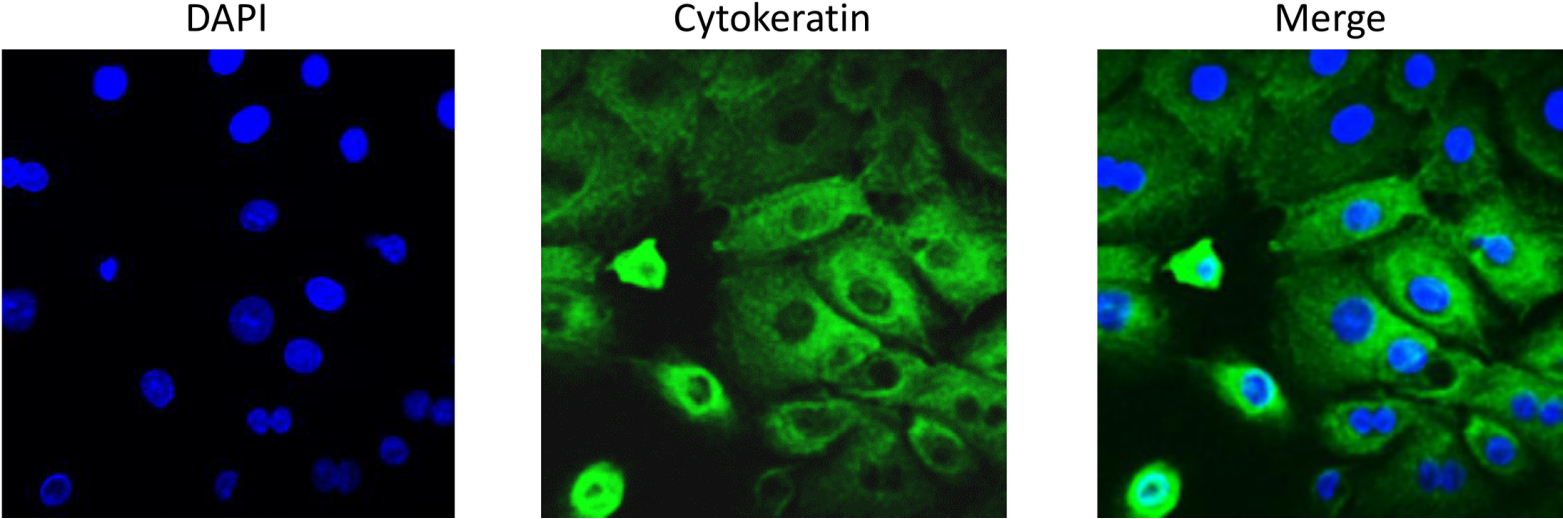
Cytokeratin staining of primary AECs. Primary AECs were fluorescently labeled for cytokeratin 18 to show cultures were predominantly epithelial cells. AEC-amnion epithelial cell.

Primary amnion cells isolated from term, not in labor C-sections were positive for cytokeratin 18, a commonly used marker for epithelial cells (Fig 1).

### Cell cycle analysis and p38 MAPK activation of control and CSE treated AECs

To determine the pattern of the cell cycle in control and CSE AECs, cell cycle analysis was performed. Table 1 summarizes the results of the cell cycle analysis. While majority of the control and CSE treated AECs were in G1 phase, noticeable differences between the two groups can be seen (Table 1). As can be seen in Table 1, the percentage of SubG0G1 phase cells was much higher in CSE treated AECs than controls. The SubG0G1 phase includes cellular debris, as well as late apoptotic and necrotic cells and suggestive of cell cycle arrest and senescence associated changes after CSE treatment. The percentage of control cells in S phase and G2 (indicating active proliferation) were almost double in control compared to CSE treated AECs showing normal progression of cell cycles in these cells. These data represent the physiologic state of the cell.

**Table 1:** Cell cycle analysis and p38 MAPK activation of control and CSE treated amnion cells.

	SubG0G1	G1	S	G2	P-p38 MAPK
**Control (untreated) AEC**	**6.9%**	**73.4%**	**15.6%**	**4.1%**	**1.8%**
**CSE Treated AEC**	**36.4%**	**53.6%**	**7.9%**	**2.1%**	**35.9%**

To further confirm this physiologic state, we also examined p38MAPK activation, an oxidative stress response indicator activated by various stressors, using flow cytometry. AECs treated with CSE showed higher p38 MAPK activation than control cells, indicating CSE AECs were undergoing oxidative stress and cellular senescence.

### Characteristics of exosomes released by primary amnion epithelial cells under control and CSE conditions

Exosomes isolated from AECs under standard (control) and oxidative (CSE) conditions were characterized using four different methods–electron microscopy (for shape and morphology), particle sizing (size), western blot and flow cytometry (specific markers and cargo contents) and Nano drop analysis (DNA quantitation).

### Exosome size and morphology from Control and CSE cells

**Fig 2:**
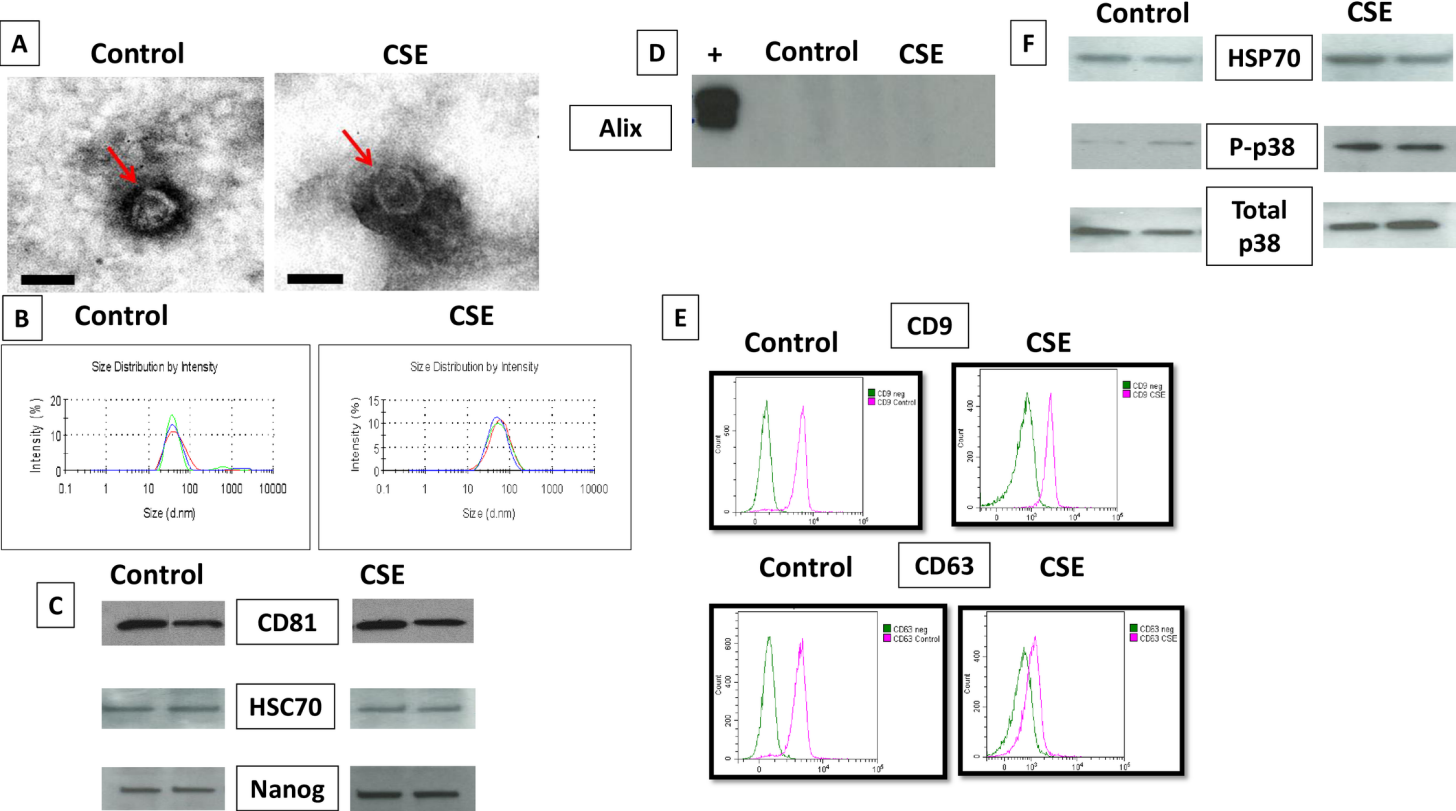
Characterization of exosomes released from amnion cells grown under standard (control) and oxidative (CSE) conditions. A: Electron microscopy showing cup-shaped vesicles that have a size distribution of 30–50 nm (arrow indicates exosomes, scale bar represents 50 nm). B: Size distribution analysis of exosomes from amnion cells untreated control (39.16 ±2.97) and CSE (45.53 ±2.34) exosomes. P<0.05 using paired sample t test. C: Western blot analysis showed the presence of exosome markers CD81 and HSC70, as well as embryonic stem cell marker, Nanog, indicating amnion epithelial cell origin. D: Western blot analysis shows lack of expression of Alix in AEC derived exosomes. E: Flow cytometric characterization of exosome tetraspanin markers exosome markers CD9 and CD63. X-axis is FITC intensity, y-axis is count, or number of beads positive for exosomes. Green represents negative control (neg). F: Western blot analysis showing differences in specific markers. Presence of stress responsive and pro-senescence marker p38 mitogen activated protein kinase (MAPK) and one of the DAMP) markers, HSP70, in exosomes from both control and CSE treated AECs. Expression of P-p38MAPK was higher in exosomes from AECs treated with CSE compared to control. AEC-amnion epithelial cell, CSE-cigarette smoke extract, DAMP-damage associated molecular pattern.

TEM analysis (Fig 2A) revealed vesicles with classic exosomal morphology. Both untreated AECs and CSE treated AECs produced cup shaped vesicles with a size distribution between 30–50 nm, which is consistent with published reports for exosomes in general, as well for exosomes from reproductive tissues [31,34,47,48]. Size distribution by intensity graphs for control and CSE treated AEC exosomes indicate the absence of larger material in each sample (Fig 2B), such as microvesicles and apoptotic bodies validating our isolation procedures and purity of exosome preparations. DLS analysis also confirmed the size distribution of 3 control and 3 CSE exosome isolations (mean ±STD) (control 39.1±2.97; CSE 45.5±2.34). CSE treated AECs produced slightly larger exosomes than exosomes from untreated AECs (p < 0.05). The number of exosomes as determined by total protein concentrations per 1 million cells were similar under both conditions. However, we acknowledge that this is not the best approach to quantitate exosomes.

### Exosome and AEC specific markers from Control and CSE cells

Western blot analysis was performed to determine exosome enriched markers and cargo contents. AEC exosomes showed the presence of markers CD81 and HSC70 (Fig 2C) regardless of treatment. We used two embryonic and amnion stem cell specific markers, Nanog (a transcription factor) and octamer-binding transcription factor 4 (Oct-4), to confirm origin of exosomes [49–54]. Although these two proteins are not exclusive to AECs, they are well documented in both amniotic epithelial and mesenchymal cells and are well reported to characterize AECs. AECs from both untreated and treated AECs demonstrated Nanog but Oct-4 was not localized in any of our exosome preparations.

We also used western blot analysis to detect the expression of Alix in AEC derived exosomes. As shown in Fig 2D, western blot analysis did not demonstrate the presence of Alix in either untreated or CSE treated AEC exosomes.

Flow cytometry was performed to further characterize the exosomes using tetraspanin markers CD9 and CD63 (Fig 2E), common markers used for exosome identification [55]. The shift in FITC intensity on the representative histograms in Fig 2E indicates beads positive for either CD9 or CD63 exosomes (pink peaks) relative to the negative control (no exosomes) (green peaks). By graphing forward scatter versus FITC intensity, we calculated the percentage of beads positive for exosome markers. After subtracting the negative control to account for background, results are expressed as percentage of beads positive for CD9 or CD63 expressing exosomes. Exosomes from control AECs showed an average of 70% for CD9 compared to 38% in CSE treated AEC exosomes. Similar decrease was also seen for CD63 (64% and 19% in controls vs CSE respectively). Difference in exosome marker expression was found to be statistically significant (p < 0.05).

### Characterization of exosomal cargo from Control and CSE treated AECs

Western blot analysis showed the presence of active forms of pro-senescence and parturition associated marker p38 MAPK (P-p38 MAPK) and one of the damage associated molecular pattern (DAMP) molecule HSP70 in exosomes from both control and CSE treated AECs. As shown in Fig 1F HSP70, P-p38 MAPK and total p38 MAPK were seen in both untreated and treated exosomes. However, the intensity of bands for P-p38 MAPK was higher in exosomes from AECs treated with CSE.

**Fig 3:**
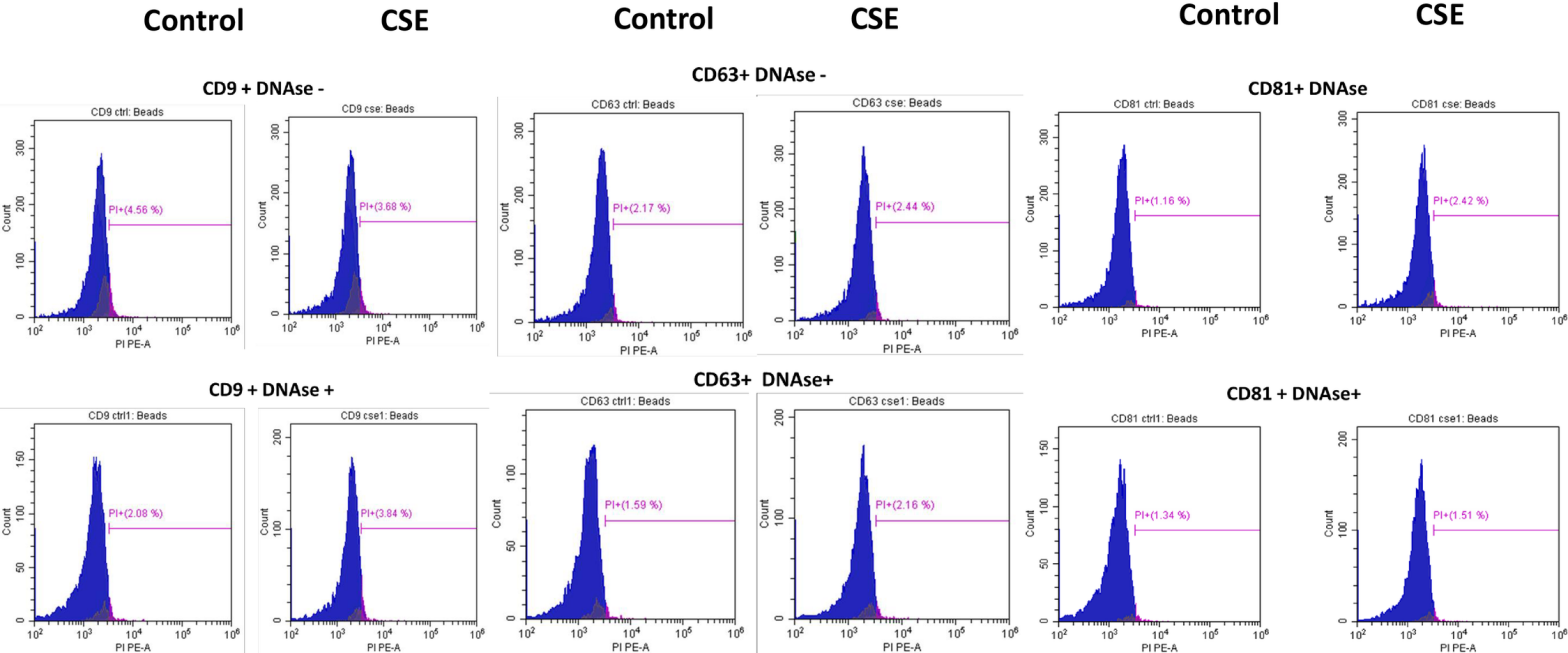
Documentation of DNA by flow cytometry in exosomes using propidium iodide (PI). There was no significant difference in DNA content in exosomes untreated with DNase (top) and those pretreated with DNase (bottom). There was also no significant difference in DNA content between CD9, CD63 and CD81 positive exosomes. The average DNA content was similar for both control (4.7 ng/μL) and CSE (5.1 ng/μL) derived exosomes. AEC-amnion epithelial cell, CSE-cigarette smoke extract.

Cell free fetal DNA are associated with human parturition through inflammatory activation [56]. Telomere fragments from fetal cells can also cause sterile inflammation in fetal tissues associated with parturition [13]. Exosomes are known to carry double and single stranded DNA fragments and cause immune activation in recipient cells [57]. Therefore, we examined untreated and CSE treated AEC derived exosomes for DNA fragments. Flow cytometry analysis for DNA content of exosomes from control and CSE treated cells (Fig 3) showed DNA within exosome preparations. There was no significant difference in DNA content between DNase treated and untreated nor between control and CSE treated AEC exosomes suggesting that external contaminant DNA are unlikely in our preparations and DNA fragmentation expected after CSE treatment did not result in increased exosomal content. We further confirmed the presence of DNA in both control and CSE exosomes by quantifying the amount of DNA using A260/280 and confirmed data obtained from flow cytometric analysis. Average DNA concentration from control exosomes was 4.7 ng/μL ± 0.794 while CSE exosomes averaged 5.1 ng/μL ± 1.86.

**Fig 4:**
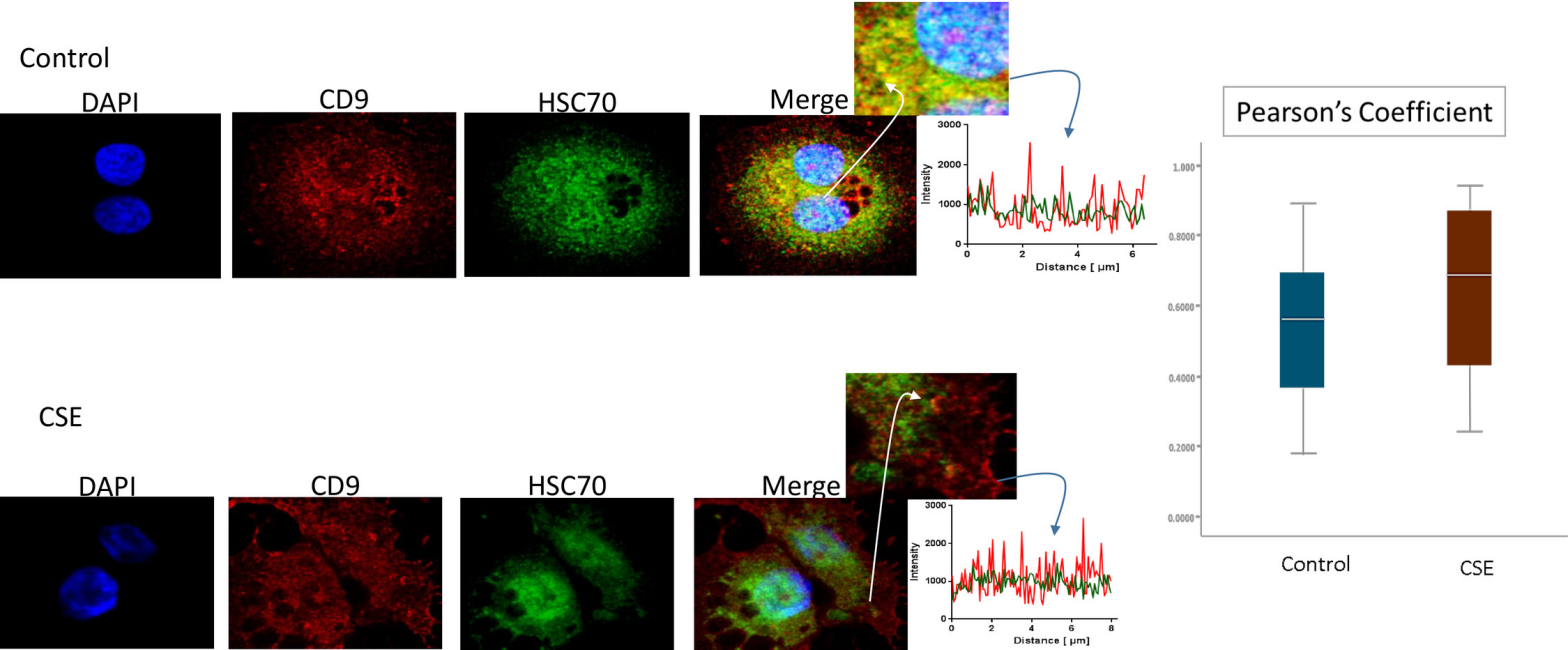
Colocalization of exosome marker HSC70 and CD9 in AECs. Control (untreated AECs) (top panel) have a similar amount of HSC70 (green) colocalization with CD9 exosome marker (red) compared to AECs exposed to CSE (bottom panel). The line graphs represent overlap between CD9 and HSC70 signal at the region of interest. The bar graph represents no significant differences between the two groups (Pearson’s Coefficient p = ns). AEC-amnion epithelial cell, CSE-cigarette smoke extract.

Immunofluorescence staining was used to colocalize markers within exosomes. As shown in Fig 4, HSC70 was colocalized with CD9 markers (Fig 4). The colocalizations were similar in both control and CSE treated AEC exosomes. This is also represented in the line graph of signal intensity over distance and bar graph representing mean Pearson’s coefficient of colocalized volume from both control and CSE AEC exosomes. These findings suggest that HSC70 is a marker of AEC derived exosomes, confirming the western blot data.

**Fig 5:**
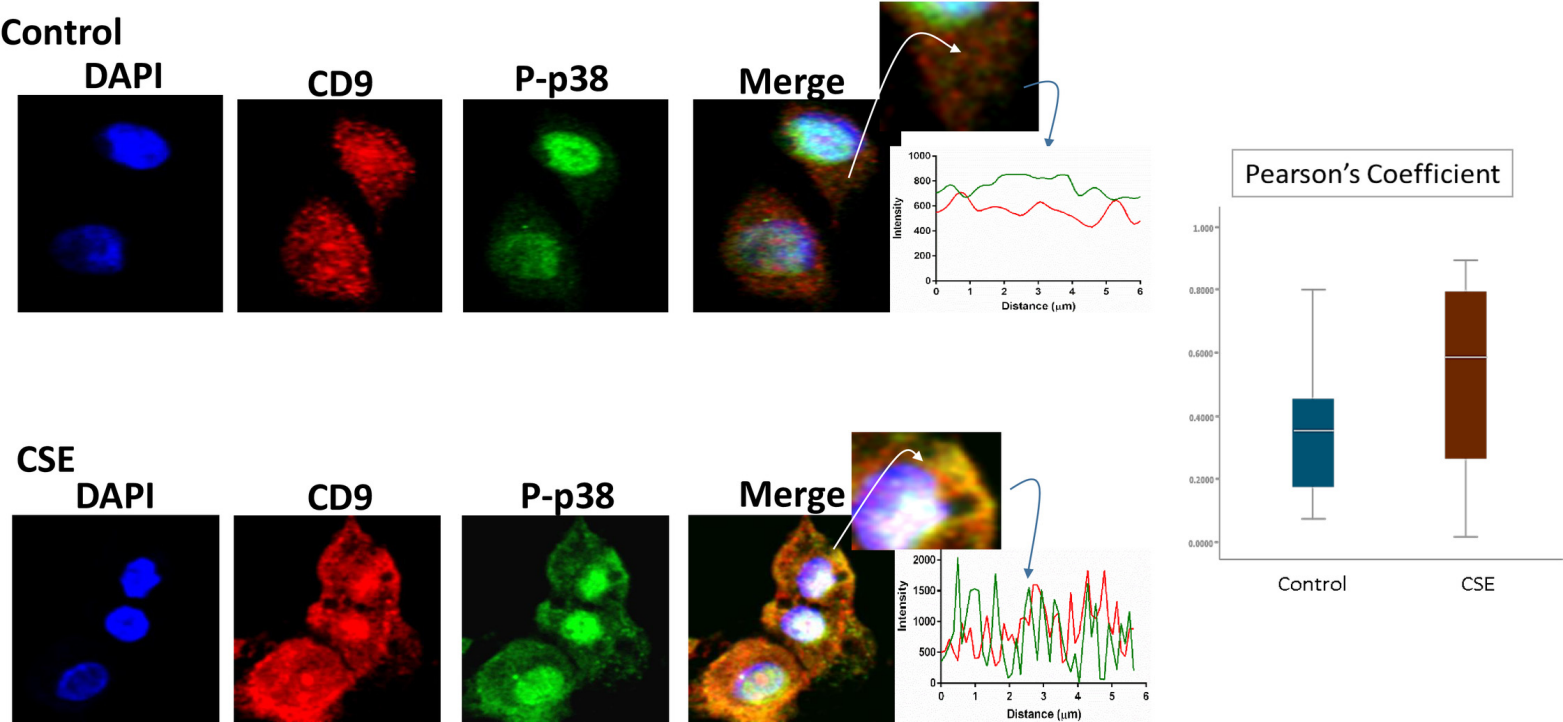
Colocalization of exosome marker H3 and CD9 in AECs. Immunofluorescence imaging of control (top panel) and CSE treated amnion cells (bottom panel) show colocalization differences of H3 (green) and CD9 (red). Significantly higher colocalization (line graph and bar graph) of H3 was seen after CSE treatment compared to control (Pearson’s Coefficient P<0.0001) AEC-amnion epithelial cell, CSE-cigarette smoke extract.

**Fig 6:**
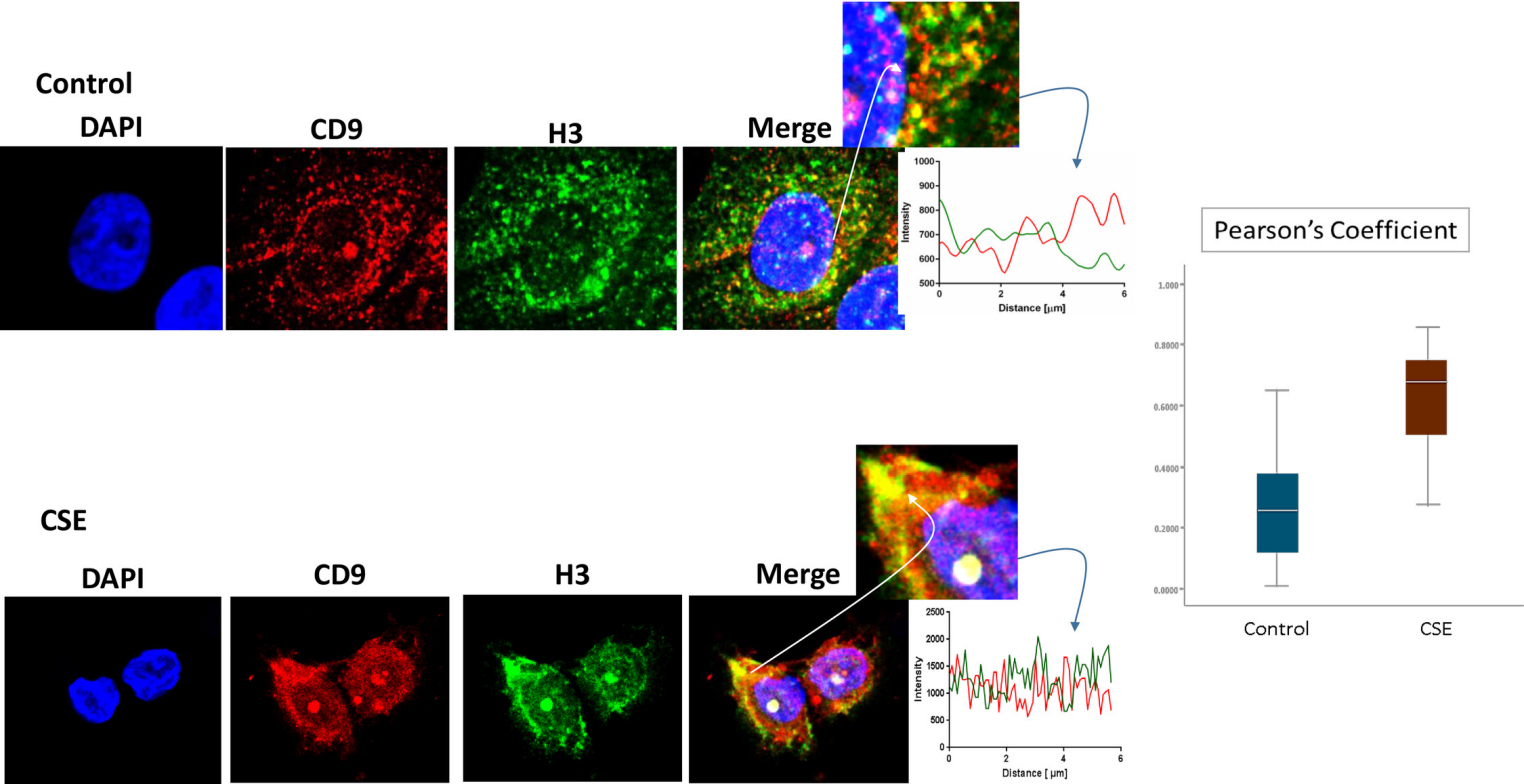
Colocalization of exosome marker HSP70 and CD9 in AECs. Colocalization of DAMP, HSP70, and CD9: Immunofluorescence imaging of control (top panel) and CSE treated amnion cells (bottom panel) show colocalization differences of HSP70 (green) and CD9 (red). Significantly higher colocalization (line graph and bar graph) of H3 was seen after CSE treatment compared to control (Pearson’s Coefficient P<0.001) AEC-amnion epithelial cell, CSE-cigarette smoke extract; DAMP–Damage associated molecular pattern.

**Fig 7:**
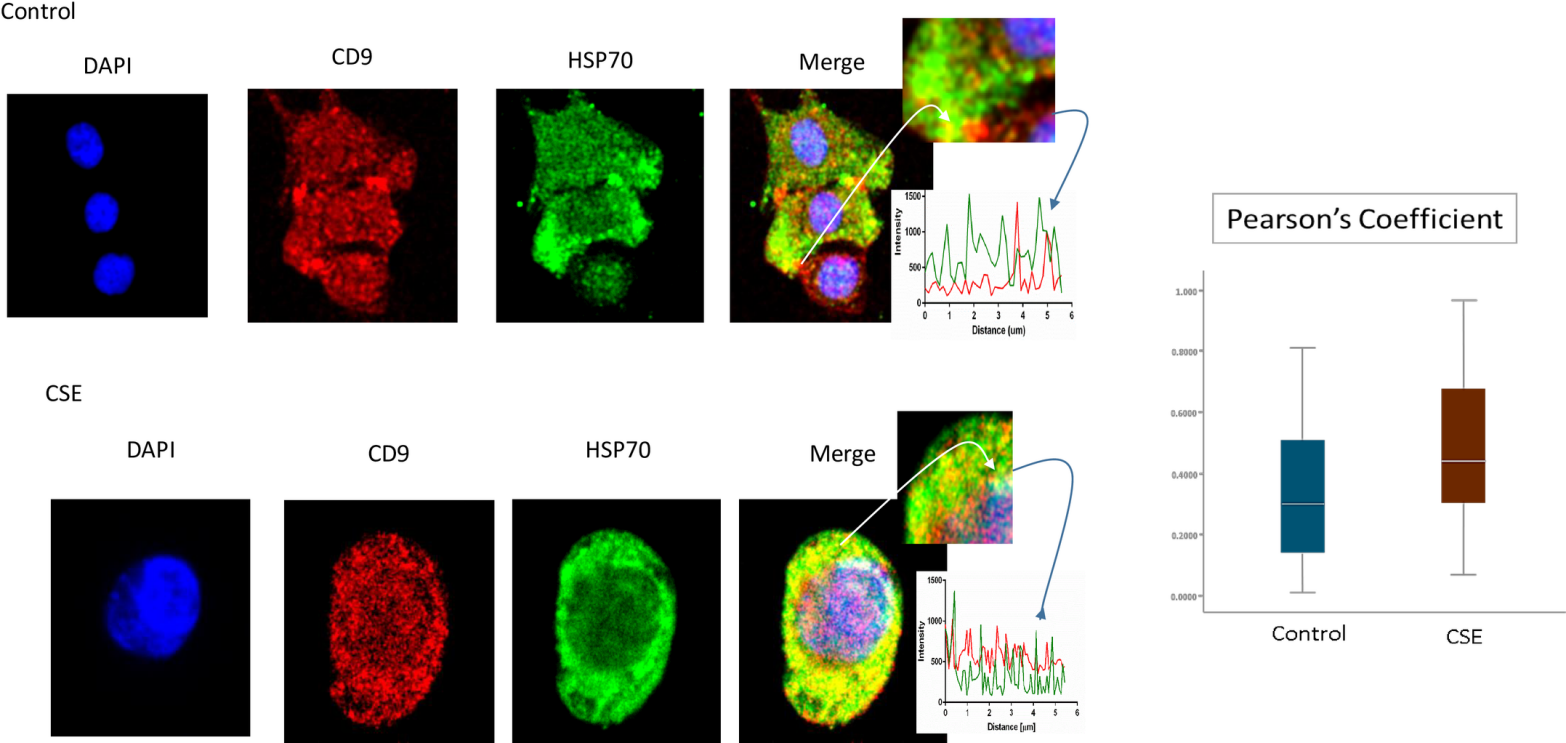
Colocalization of exosome marker P-p38MAPK and CD9 in AECs. Colocalization of pro-senescence marker P-p38MAPK and CD9: Immunofluorescence imaging of control (top panel) and CSE treated amnion cells (bottom panel) show colocalization differences of HSP70 (green) and CD9 (red). Significantly higher colocalization (line graph and bar graph) of H3 was seen after CSE treatment compared to control (Pearson’s Coefficient P<0.0001) AEC-amnion epithelial cell, CSE-cigarette smoke extract.

P-p38 MAPK (Fig 5), Histone 3 (H3, Fig 6), and HSP70 (Fig 7) were colocalized with exosome marker CD9 using immunofluorescent microscopy to determine whether exosomes reflect the physiologic state of AECs. All studied markers are associated with oxidative stress response and activation of p38 MAPK in amnion cells, which is the signaler in senescence response to CSE. H3 and HSP70 are p38 MAPK responder genes in stress associated cellular signaling [58,59]. Colocalization was quantified using Pearson’s correlation coefficient and graphed comparing control and CSE mean values. Colocalization of all three markers were significantly higher in CSE treated AEC exosomes compared to control AEC exosomes (mean Pearson’s correlation coefficients for each were statistically significant (p<0.05)) confirming CSE causes increased cargo of these markers by exosomes. CSE induced oxidative stress damage leads to senescence of AEC through p38 MAPK signaling (12] and current data confirm that P-p38 MAPK and its responder proteins H3 and HSP70 can also get packaged inside exosomes at a higher level reflecting the physiologic state of AECs.

### Proteomic analysis of CTs-derived exosomes

**Fig 8:**
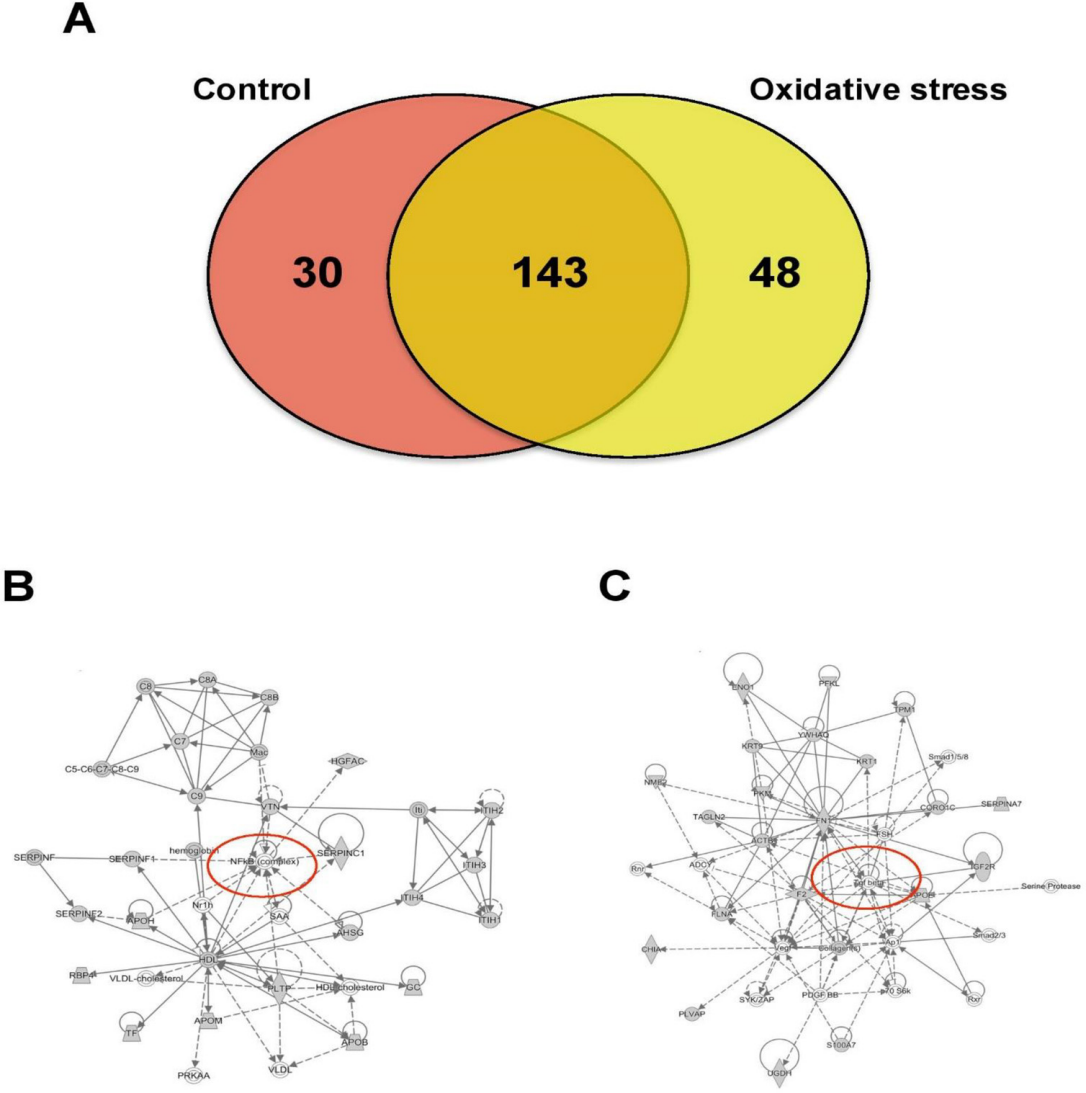
Proteomic analysis of AEC-derived exosome proteins. (A) The Venn diagram represents the distribution of common and unique proteins identified by nanospray LC-MS/MS in exosomes released from AEC cultured under normal or stress conditions. List contain 221 unique proteins. (B and C) Proteins identified in exosomes isolated from AEC under normal (B) or oxidative stress (C) conditions were submitted to IPA network analysis. Red circle: central molecules involved in the signaling pathways.

**Fig 9:**
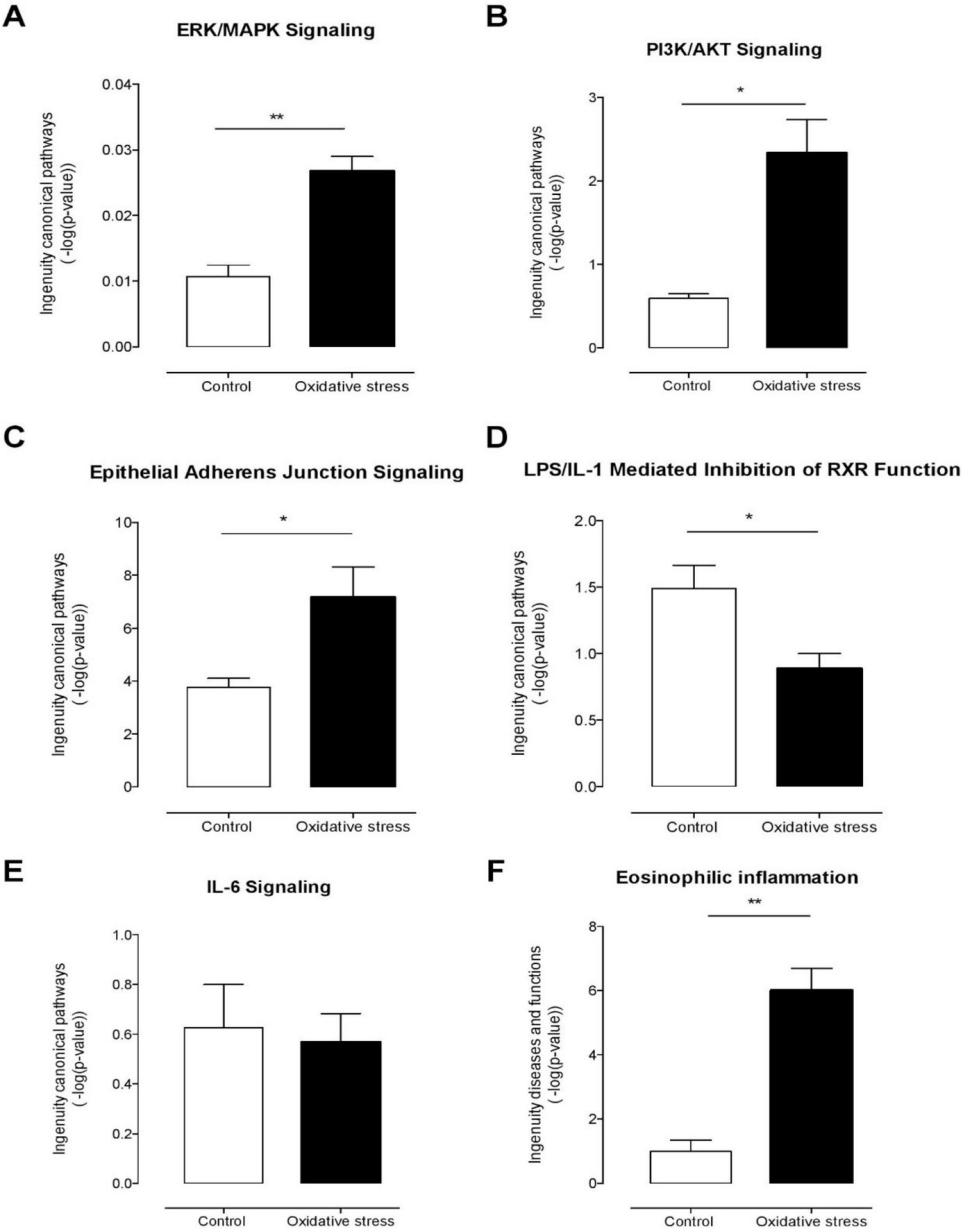
Ingenuity pathway analysis of AEC-derived-exosomes proteins. Exosomal protein identified under normal (control) or oxidative stress conditions were analyzed using the IPA software. Comparison of canonical pathways: (A) ERK/MAPK, (B) PI3K/AKT, (C) epithelial adherens junctions, (D) LPS/IL-1 mediated inhibition of RXR function and (E) IL-6 signaling. Diseases and functions analysis: (F) eosinophilic inflammation. Values are mean ± SD. In A and F, **p < 0.001. In B, C and D, *p < 0.005.

Mass spectrometry analysis identified over 200 exosomal proteins (S1 Table). We also identified unique proteins for each condition (Fig 8A; Tables 2 and 3). The number of proteins identified in exosomes isolated from AEC exposed to CSE was higher (193) compared to control (173). We investigated the molecular network that can be activated by the proteins identified in exosomes isolated from AEC cultured under normal (8B) and oxidative stress conditions (8C). Interestingly, NF-κβ complex seems to be a central regulator in the molecular network that can be activated by exosomes from AEC cultured under normal conditions, where TGF-β might regulate the molecular network from exosomes from AEC treated with CSE. We have already reported that CSE treatment produce minimal activation of NF-κB compared to control and the exosomal proteomic analysis further supports our earlier findings [60]. We have also seen evidence of epithelial-mesenchymal transition (EMT) of amnion epithelial cells under oxidative stress conditions. TGF-β is a major mediator of EMT [61–65]. Molecular networks in CSE treated exosomes confirms that TGF-β mediated EMT may be functional in AECs under oxidative stress. Using Ingenuity Pathway Analysis (IPA), a bioinformatics approach, we examined the biological pathways represented by differentially expressed proteins from our proteomic analysis. The canonical pathways determined by exosomal cargo contents showed ERK/MAPK (Fig 9A), PI3K/AKT (Fig 9B) and epithelial adherent junctions (Fig 9C) was significantly higher in exosomes from AEC under oxidative stress conditions compared to the control. On the other hand, canonical pathways as LPS/IL-1 mediated inhibition of the nuclear receptor retinoid X receptor (RXR) function and IL-6 signaling were significantly lower and unchanged in exosomes from AEC cultured under oxidative stress conditions compared to control, respectively. Finally, analysis of diseases and functions showed that exosomes isolated from AEC under oxidative stress conditions might significantly increase the eosinophilic inflammation compared to control. Interestingly, higher amount of eosinophil cells in the amniotic fluids has been associated with preterm labor [66].

**Table 2:** Proteomics analysis of exosomal cargo identified 30 unique markers in exosomes derived from amnion epithelial cells grown in normal culture conditions.

ID	Symbol	Entrez Gene Name	Function	Location
**ADK_HUMAN**	**ADK**	**adenosine kinase**	**metabolism**	**Nucleus**
**AK1A1_HUMAN**	**AKR1A1**	**aldo-keto reductase family 1, member A1 (aldehyde reductase)**	**metabolism**	**Cytoplasm**
**ASSY_HUMAN**	**ASS1**	**argininosuccinate synthase 1**	**metabolism**	**Cytoplasm**
**CADH5_HUMAN**	**CDH5**	**cadherin 5**	**cell adhesion**	**Plasma Membrane**
**COBA1_HUMAN**	**COL11A1**	**collagen, type XI, alpha 1**	**cell adhesion**	**Extracellular Space**
**COR1A_HUMAN**	**CORO1A**	**coronin 1A**	**structural**	**Cytoplasm**
**DYH8_HUMAN**	**DNAH8**	**dynein, axonemal, heavy chain 8**	**structural**	**Cytoplasm**
**DESP_HUMAN**	**DSP**	**desmoplakin**	**cell adhesion**	**Plasma Membrane**
**URP2_HUMAN**	**FERMT3**	**fermitin family member 3**	**cell adhesion**	**Cytoplasm**
**FMOD_HUMAN**	**FMOD**	**fibromodulin**	**structural**	**Extracellular Space**
**GSTA2_HUMAN**	**GSTA2**	**glutathione S-transferase alpha 2**	**metabolism**	**Cytoplasm**
**HGD_HUMAN**	**HGD**	**homogentisate 1,2-dioxygenase**	**metabolism**	**Cytoplasm**
**HORN_HUMAN**	**HRNR**	**hornerin**	**structural**	**Cytoplasm**
**IBP7_HUMAN**	**IGFBP7**	**insulin like growth factor binding protein 7**	**cell adhesion**	**Extracellular Space**
**KPRP_HUMAN**	**KPRP**	**keratinocyte proline-rich protein**	**other**	**Cytoplasm**
**LYSC_HUMAN**	**LYZ**	**lysozyme**	**immune response**	**Extracellular Space**
**MAOX_HUMAN**	**ME1**	**malic enzyme 1, NADP(+)-dependent, cytosolic**	**metabolism**	**Cytoplasm**
**NCAM1_HUMAN**	**NCAM1**	**neural cell adhesion molecule 1**	**cell adhesion**	**Plasma Membrane**
**NELL2_HUMAN**	**NELL2**	**neural EGFL like 2**	**cell adhesion**	**Extracellular Space**
**PARVA_HUMAN**	**PARVA**	**parvin alpha**	**cell adhesion**	**Cytoplasm**
**PROF1_HUMAN**	**PFN1**	**profilin 1**	**cell adhesion**	**Cytoplasm**
**PROS_HUMAN**	**PROS1**	**protein S (alpha)**	**cell migration**	**Extracellular Space**
**PYGB_HUMAN**	**PYGB**	**phosphorylase, glycogen; brain**	**metabolism**	**Cytoplasm**
**S10A8_HUMAN**	**S100A8**	**S100 calcium binding protein A8**	**inflammation**	**Cytoplasm**
**HEP2_HUMAN**	**SERPIND1**	**serpin peptidase inhibitor, clade D (heparin cofactor), member 1**	**cell migration**	**Extracellular Space**
**SHBG_HUMAN**	**SHBG**	**sex hormone-binding globulin**	**transport**	**Extracellular Space**
**SPRR3_HUMAN**	**SPRR3**	**small proline-rich protein 3**	**structural**	**Cytoplasm**
**ST1E1_HUMAN**	**SULT1E1**	**sulfotransferase family 1E member 1**	**metabolism**	**Cytoplasm**
**VCAM1_HUMAN**	**VCAM1**	**vascular cell adhesion molecule 1**	**cell adhesion**	**Plasma Membrane**
**VNN1_HUMAN**	**VNN1**	**vanin 1**	**cell adhesion**	**Plasma Membrane**

**Table 3:** Proteomics analysis of exosomal cargo identified 48 unique markers in exosomes derived from amnion epithelial cells grown under oxidative stress conditions.

ID	Symbol	Entrez Gene Name	Function (UniProt)	Location
**ACES_HUMAN**	**ACHE**	**acetylcholinesterase (Yt blood group)**	**signaling**	**Plasma Membrane**
**ACTN1_HUMAN**	**ACTN1**	**actinin, alpha 1**	**structural**	**Cytoplasm**
**AOC3_HUMAN**	**AOC3**	**amine oxidase, copper containing 3**	**cell adhesion**	**Plasma Membrane**
**APOA4_HUMAN**	**APOA4**	**apolipoprotein A-IV**	**binding protein**	**Extracellular Space**
**RIMB1_HUMAN**	**BZRAP1**	**benzodiazepine receptor (peripheral) associated protein 1**	**binding protein**	**Cytoplasm**
**CR063_HUMAN**	**C18orf63**	**chromosome 18 open reading frame 63**	**Other**	**Other**
**CO2_HUMAN**	**C2**	**complement component 2**	**immune response**	**Extracellular Space**
**CAB39_HUMAN**	**CAB39**	**calcium binding protein 39**	**cell cycle**	**Cytoplasm**
**CADH6_HUMAN**	**CDH6**	**cadherin 6**	**cell adhesion**	**Plasma Membrane**
**CFAB_HUMAN**	**CFB**	**complement factor B**	**immune response**	**Extracellular Space**
**CNDP2_HUMAN**	**CNDP2**	**CNDP dipeptidase 2 (metallopeptidase M20 family)**	**cell cycle**	**Cytoplasm**
**COR1C_HUMAN**	**CORO1C**	**coronin 1C**	**signaling**	**Cytoplasm**
**DPYS_HUMAN**	**DPYS**	**dihydropyrimidinase**	**metabolism**	**Cytoplasm**
**FILA_HUMAN**	**FLG**	**filaggrin**	**structural**	**Cytoplasm**
**FILA2_HUMAN**	**FLG2**	**filaggrin family member 2**	**structural**	**Cytoplasm**
**GDIB_HUMAN**	**GDI2**	**GDP dissociation inhibitor 2**	**signaling**	**Cytoplasm**
**GSTM5_HUMAN**	**GSTM5**	**glutathione S-transferase mu 5**	**metabolism**	**Cytoplasm**
**HS90B_HUMAN**	**HSP90AB1**	**heat shock protein 90kDa alpha family class B member 1**	**inflammation**	**Cytoplasm**
**IGF2_HUMAN**	**IGF2**	**insulin like growth factor 2**	**signaling**	**Extracellular Space**
**ITA2_HUMAN**	**ITGA2**	**integrin subunit alpha 2**	**cell adhesion**	**Plasma Membrane**
**LAMC1_HUMAN**	**LAMC1**	**laminin subunit gamma 1**	**cell adhesion**	**Extracellular Space**
**MYH2_HUMAN**	**MYH2**	**myosin, heavy chain 2, skeletal muscle, adult**	**structural**	**Cytoplasm**
**NDKB_HUMAN**	**NME2**	**NME/NM23 nucleoside diphosphate kinase 2**	**metabolism**	**Nucleus**
**PCLO_HUMAN**	**PCLO**	**piccolo presynaptic cytomatrix protein**	**signaling**	**Cytoplasm**
**PFKAL_HUMAN**	**PFKL**	**phosphofructokinase, liver**	**metabolism**	**Cytoplasm**
**PAFA_HUMAN**	**PLA2G7**	**phospholipase A2 group VII**	**inflammation**	**Extracellular Space**
**PLVAP_HUMAN**	**PLVAP**	**plasmalemma vesicle associated protein**	**signaling**	**Plasma Membrane**
**PPIB_HUMAN**	**PPIB**	**peptidylprolyl isomerase B**	**structural**	**Cytoplasm**
**PRDX1_HUMAN**	**PRDX1**	**peroxiredoxin 1**	**inflammation**	**Cytoplasm**
**PRDX2_HUMAN**	**PRDX2**	**peroxiredoxin 2**	**inflammation**	**Cytoplasm**
**PSA1_HUMAN**	**PSMA1**	**proteasome subunit alpha 1**	**cell cycle**	**Cytoplasm**
**PSA4_HUMAN**	**PSMA4**	**proteasome subunit alpha 4**	**cell cycle**	**Cytoplasm**
**PSA6_HUMAN**	**PSMA6**	**proteasome subunit alpha 6**	**cell cycle**	**Cytoplasm**
**PSA7_HUMAN**	**PSMA7**	**proteasome subunit alpha 7**	**cell cycle**	**Cytoplasm**
**PSMD5_HUMAN**	**PSMD5**	**proteasome 26S subunit, non-ATPase 5**	**cell cycle**	**Other**
**PYGL_HUMAN**	**PYGL**	**phosphorylase, glycogen, liver**	**metabolism**	**Cytoplasm**
**RAN_HUMAN**	**RAN**	**RAN, member RAS oncogene family**	**cell cycle**	**Nucleus**
**RAP1B_HUMAN**	**RAP1B**	**RAP1B, member of RAS oncogene family**	**transport**	**Cytoplasm**
**S10A7_HUMAN**	**S100A7**	**S100 calcium binding protein A7**	**inflammation**	**Cytoplasm**
**S10A9_HUMAN**	**S100A9**	**S100 calcium binding protein A9**	**inflammation**	**Cytoplasm**
**TAGL2_HUMAN**	**TAGLN2**	**transgelin 2**	**binding**	**Cytoplasm**
**TFR1_HUMAN**	**TFRC**	**transferrin receptor**	**transport**	**Plasma Membrane**
**TENX_HUMAN**	**TNXB**	**tenascin XB**	**cell adhesion**	**Extracellular Space**
**TPM1_HUMAN**	**TPM1**	**tropomyosin 1 (alpha)**	**structural**	**Cytoplasm**
**TBA4A_HUMAN**	**TUBA4A**	**tubulin alpha 4a**	**structural**	**Cytoplasm**
**RL40_HUMAN**	**UBA52**	**ubiquitin A-52 residue ribosomal protein fusion product 1**	**inflammation**	**Cytoplasm**
**WDR1_HUMAN**	**WDR1**	**WD repeat domain 1**	**transport**	**Extracellular Space**
**1433T_HUMAN**	**YWHAQ**	**tyrosine 3-monooxygenase/tryptophan 5-monooxygenase activation protein, theta**	**signaling**	**Cytoplasm**

## Discussion

Ongoing studies suggest the development of fetal membrane senescence as a mechanism associated with term parturition [9,12,14]. Oxidative stress, antioxidant depletion, oxidative stress induced senescence, stress associated p38 MAPK activation, and sterile inflammation, are associated with normal term human parturition [9,12,67,68]. These findings in *in vivo* clinical samples were recapitulated in vitro using normal term not in labor fetal membrane explant cultures or AECs where oxidative stress induced transition of fetal membranes to a senescence phenotype mimicked term labor status [12–14]. This suggests that exogenous oxidative stress at term promotes senescence and senescent fetal membrane cells to signal parturition by enhancing the overall inflammatory load in the uterine cavity. In this study, we demonstrated that AEC-derived exosomes may serve as carriers of signals of communication between various tissue layers by senescent fetal cells. Exosomes, generated as a consequence of multivesicular endosome (MVE) fusion with the plasma membranes [69–71], and their contents (protein, DNA, and all forms of RNAs), represent the character and physiologic state of the cell of origin that makes them good vectors of paracrine signaling.

The primary aim of this study was to isolate, characterize and demonstrate that AEC derived exosomes reflect the physiological status of the cells of origin. Our key findings are as follows: 1) AEC derived exosomes demonstrated classic shape, size and markers (CD9, 63, 81, HSC 70) along with amnion cell-stem cell specific transcription factor Nanog, regardless of treatment. 2) AEC derived exosomes do not show the presence of ESCRT-associated protein Alix and amnion cell stem cell marker Oct-4 in their cargo. 3) CSE treatment caused increased colocalization of H3, HSP70 and active p38 (P-p38) MAPK in AEC exosomes. Increased localization of these proteins demonstrated a pathophysiological phenotype of AECs in response to CSE induced oxidative stress. Although the functional relevance is unclear, this is the first report to demonstrate P-p38 MAPK as an exosomal cargo. We propose that AEC-derived exosomes demonstrate the characteristics of a pathophysiological state of the cell of origin and that the presence of P-p38 MAPK, a marker of inflammation, is a key mediator of senescence induction and sterile inflammation. Our studies suggest the importance of AEC derived exosome cargo in causing potential functional changes in feto-maternal compartments. Although we do not report any functional role of AEC exosomes during pregnancy, ongoing research is focused on determining such a role.

The impact of p38 MAPK activation is well documented in fetal tissues but no data exist on its functional contributions on myometrial side whose contractility determines pregnancy outcome. Functional progesterone withdrawal and subsequent myometrial activation (i.e. increased contractility and excitability) are key events in the initiation of labor [72–75]. Functional progesterone withdrawal, which is mediated, in part, by switching of the PR-A:PR-B ratio in myometrial cells from PR-B-dominance (mediates anti-inflammatory and relaxing actions of progesterone) to PR-A-dominance (inhibits the anti-inflammatory actions of progesterone and increase contractility) [72–75]. A key finding in breast cancer cell lines is that the PR-A: PR-B ratio is determined by the stability of the PR-A and PR-B proteins, which is caused by post-translational modifications, especially phosphorylation by MAPKs at specific serine residues in the N-terminal domain [76–79]. Khan et al found that PR-A stability is increased by MEKK1-induced p38 MAPK activation, which increased the PR-A: PR-B ratio; whereas PR-B stability was increased by activation of ERK1/2, leading to a decrease in the PR-A: PR-B ratio. Our unpublished data (performed in collaboration with Dr. Sam Mesiano) in myometrial cells demonstrate that CSE can cause functional progesterone withdrawal in myocytes through p38 MAPK mediated mechanism, a process reversed by p38 MAPK inhibitor SB203580. This determined the impact of p38 MAPK in human parturition. Therefore exosomal transport of fetal cell derived P-p38 MAPK to the maternal side may be influential in determining the status of pregnancy. This is also dependent on the number of exosomes and load of p38 MAPK that can reach the maternal side. Exosomes may carry other inflammatory molecules (SASP) from senescent fetal cells and they may also enhance the inflammatory load on the maternal side. We postulate that based on the physiologic state of cell, exosome cargo and signaling may determine the outcome of pregnancy. Placental derived exosomes have been well documented in maternal liquid biopsies and their changes (quantity and contents) have been implicated in various pregnancy associated pathologies [31–37,40,42,46–48,80–94].

Although AEC exosomes demonstrated the consistent presence of classical exosomal markers and the amnion stem cell marker Nanog, we did not show one of the ESCRT class of proteins, Alix. ESCRT complex dependent and independent mechanisms of exosomal assembly and secretion have been described as a tissue/cell specific physiological phenomenon [27]. Based on our experimental approaches, AEC exosomes are likely derived without the participation of the ESCRT protein; however, we do not rule out mediation by other classes of ESCRT proteins. We have documented three of the tetraspanin protein markers (CD9, CD63 and CD81) that can participate in exosomal assembly and release of cargo.

**Fig 10:**
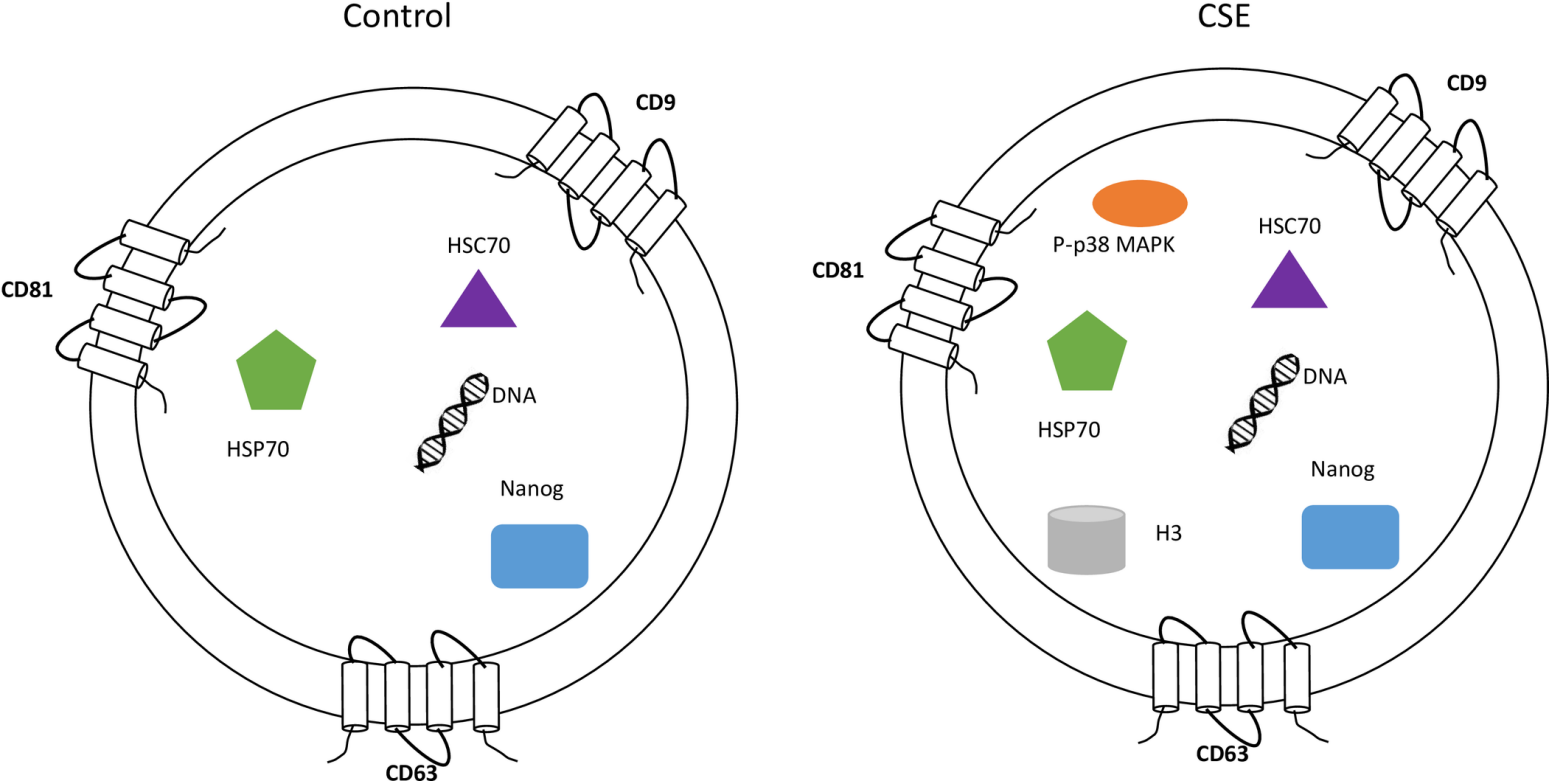
Characterization of AEC exosomes. The exosomes isolated from untreated (control) and CSE treated primary AEC carry cargo representative of the state of the origin cell. AEC exosomes contain tetraspnins (CD9, CD63 and CD81), Nanog, HSC70, HSP70 and DNA regardless of treatment, while CSE exosomes contain significantly increased amounts of P-p38MAPK and H3 compared with control exosomes.

In summary, we have demonstrated AECs produce exosomes and that their cargo reflects the status of the cell (Fig 10). Furthermore, we identified active p38 MAPK as one of the cargo proteins in exosomes derived from oxidative stress-treated AECs. Our studies highlight the significance of AEC derived exosomes as donors of p38 MAPK which plays a major role in determining the fate of pregnancy. This study demonstrated a limited number of markers and further characterization of AEC exosome cargo using proteomic approaches are warranted to elucidate the functional role of exosomes in human parturition and feto-maternal communication.

## Supporting Information

S1 Table Proteomics analysis of exosomal cargo identified over 200 proteins in exosomes derived from amnion epithelial cells grown under control and oxidative stress conditions. (DOCX)
