# Supplementary material for: Amnion-Epithelial-Cell-Derived Exosomes Demonstrate Physiologic State of Cell under Oxidative Stress
Source: PLoS One. 2016 Jun 22;11(6):e0157614. doi: 10.1371/journal.pone.0157614 (PMC4917104; doi:10.1371/journal.pone.0157614)
Supplement: S1 Table — (DOCX) [file pone.0157614.s001.docx]

| Table S1. List of proteins identified in exosomes from AEC cultured under normal (control) or oxidative stress conditions (treatment). | | | |
| --- | --- | --- | --- |
| Control | | | |
| ID | Entrez Gene Name | Location | Type(s) |
| A2MG_HUMAN | alpha-2-macroglobulin | Extracellular Space | transporter |
| ACTB_HUMAN | actin, beta | Cytoplasm | other |
| ACTN4_HUMAN | actinin, alpha 4 | Cytoplasm | transcription regulator |
| ATS13_HUMAN | ADAM metallopeptidase with thrombospondin type 1 motif 13 | Extracellular Space | peptidase |
| ADHX_HUMAN | alcohol dehydrogenase 5 (class III), chi polypeptide | Cytoplasm | enzyme |
| ADK_HUMAN | adenosine kinase | Nucleus | kinase |
| AFAM_HUMAN | afamin | Extracellular Space | transporter |
| FETA_HUMAN | alpha fetoprotein | Extracellular Space | transporter |
| SAHH_HUMAN | adenosylhomocysteinase | Cytoplasm | enzyme |
| FETUA_HUMAN | alpha-2-HS-glycoprotein | Extracellular Space | other |
| AK1A1_HUMAN | aldo-keto reductase family 1, member A1 (aldehyde reductase) | Cytoplasm | enzyme |
| AL9A1_HUMAN | aldehyde dehydrogenase 9 family member A1 | Cytoplasm | enzyme |
| ALDOA_HUMAN | aldolase, fructose-bisphosphate A | Cytoplasm | enzyme |
| AMYP_HUMAN | amylase, alpha 2A (pancreatic) | Extracellular Space | enzyme |
| ANXA1_HUMAN | annexin A1 | Plasma Membrane | enzyme |
| ANXA2_HUMAN | annexin A2 | Plasma Membrane | other |
| APOA1_HUMAN | apolipoprotein A-I | Extracellular Space | transporter |
| APOB_HUMAN | apolipoprotein B | Extracellular Space | transporter |
| APOE_HUMAN | apolipoprotein E | Extracellular Space | transporter |
| APOH_HUMAN | apolipoprotein H | Extracellular Space | transporter |
| APOM_HUMAN | apolipoprotein M | Plasma Membrane | transporter |
| ASSY_HUMAN | argininosuccinate synthase 1 | Cytoplasm | enzyme |
| BHMT1_HUMAN | betaine--homocysteine S-methyltransferase | Cytoplasm | enzyme |
| C1QT3_HUMAN | C1q and tumor necrosis factor related protein 3 | Extracellular Space | other |
| CO3_HUMAN | complement component 3 | Extracellular Space | peptidase |
| CO4A_HUMAN | complement component 4B (Chido blood group) | Extracellular Space | other |
| CO5_HUMAN | complement component 5 | Extracellular Space | cytokine |
| CO7_HUMAN | complement component 7 | Extracellular Space | other |
| CO8A_HUMAN | complement component 8, alpha polypeptide | Extracellular Space | other |
| CO8B_HUMAN | complement component 8, beta polypeptide | Extracellular Space | other |
| CO9_HUMAN | complement component 9 | Extracellular Space | other |
| CAP1_HUMAN | CAP, adenylate cyclase-associated protein 1 (yeast) | Plasma Membrane | other |
| CCD80_HUMAN | coiled-coil domain containing 80 | Nucleus | other |
| CD109_HUMAN | CD109 molecule | Plasma Membrane | other |
| CADH5_HUMAN | cadherin 5 | Plasma Membrane | other |
| CFAI_HUMAN | complement factor I | Extracellular Space | peptidase |
| COF1_HUMAN | cofilin 1 (non-muscle) | Nucleus | other |
| CHIA_HUMAN | chitinase, acidic | Extracellular Space | enzyme |
| CLC11_HUMAN | C-type lectin domain family 11 member A | Extracellular Space | growth factor |
| TETN_HUMAN | C-type lectin domain family 3 member B | Extracellular Space | other |
| CLUS_HUMAN | clusterin | Cytoplasm | other |
| CNTN1_HUMAN | contactin 1 | Plasma Membrane | enzyme |
| COAA1_HUMAN | collagen, type X, alpha 1 | Extracellular Space | other |
| COBA1_HUMAN | collagen, type XI, alpha 1 | Extracellular Space | other |
| CO1A1_HUMAN | collagen, type I, alpha 1 | Extracellular Space | other |
| CO1A2_HUMAN | collagen, type I, alpha 2 | Extracellular Space | other |
| CO2A1_HUMAN | collagen, type II, alpha 1 | Extracellular Space | other |
| CO3A1_HUMAN | collagen, type III, alpha 1 | Extracellular Space | other |
| CO5A1_HUMAN | collagen, type V, alpha 1 | Extracellular Space | other |
| CO6A1_HUMAN | collagen, type VI, alpha 1 | Extracellular Space | other |
| CO6A3_HUMAN | collagen, type VI, alpha 3 | Extracellular Space | other |
| COMP_HUMAN | cartilage oligomeric matrix protein | Extracellular Space | other |
| COR1A_HUMAN | coronin 1A | Cytoplasm | other |
| CERU_HUMAN | ceruloplasmin (ferroxidase) | Extracellular Space | enzyme |
| CBPN_HUMAN | carboxypeptidase N subunit 1 | Extracellular Space | peptidase |
| DYH8_HUMAN | dynein, axonemal, heavy chain 8 | Cytoplasm | enzyme |
| DESP_HUMAN | desmoplakin | Plasma Membrane | other |
| EF2_HUMAN | eukaryotic translation elongation factor 2 | Cytoplasm | translation regulator |
| FBLN3_HUMAN | EGF containing fibulin-like extracellular matrix protein 1 | Extracellular Space | enzyme |
| IF4A2_HUMAN | eukaryotic translation initiation factor 4A2 | Cytoplasm | translation regulator |
| ENOA_HUMAN | enolase 1, (alpha) | Cytoplasm | enzyme |
| EZRI_HUMAN | ezrin | Plasma Membrane | other |
| FA10_HUMAN | coagulation factor X | Extracellular Space | peptidase |
| FA11_HUMAN | coagulation factor XI | Extracellular Space | peptidase |
| F13A_HUMAN | coagulation factor XIII, A1 polypeptide | Extracellular Space | enzyme |
| THRB_HUMAN | coagulation factor II, thrombin | Extracellular Space | peptidase |
| FA5_HUMAN | coagulation factor V | Plasma Membrane | enzyme |
| SEPR_HUMAN | fibroblast activation protein alpha | Cytoplasm | peptidase |
| FBLN1_HUMAN | fibulin 1 | Extracellular Space | other |
| FBN1_HUMAN | fibrillin 1 | Extracellular Space | other |
| URP2_HUMAN | fermitin family member 3 | Cytoplasm | enzyme |
| FIBB_HUMAN | fibrinogen beta chain | Extracellular Space | other |
| FIBG_HUMAN | fibrinogen gamma chain | Extracellular Space | other |
| FLNA_HUMAN | filamin A | Cytoplasm | other |
| FMOD_HUMAN | fibromodulin | Extracellular Space | other |
| FINC_HUMAN | fibronectin 1 | Extracellular Space | enzyme |
| G3P_HUMAN | glyceraldehyde-3-phosphate dehydrogenase | Cytoplasm | enzyme |
| VTDB_HUMAN | group-specific component (vitamin D binding protein) | Extracellular Space | transporter |
| GPX3_HUMAN | glutathione peroxidase 3 | Extracellular Space | enzyme |
| GELS_HUMAN | gelsolin | Extracellular Space | other |
| GSTA2_HUMAN | glutathione S-transferase alpha 2 | Cytoplasm | enzyme |
| HBD_HUMAN | hemoglobin subunit delta | Other | transporter |
| HGD_HUMAN | homogentisate 1,2-dioxygenase | Cytoplasm | enzyme |
| HGFA_HUMAN | HGF activator | Extracellular Space | peptidase |
| HORN_HUMAN | hornerin | Cytoplasm | other |
| HSP7C_HUMAN | heat shock protein family A (Hsp70) member 8 | Cytoplasm | enzyme |
| MPRI_HUMAN | insulin like growth factor 2 receptor | Plasma Membrane | transmembrane receptor |
| IBP2_HUMAN | insulin like growth factor binding protein 2 | Extracellular Space | other |
| IBP7_HUMAN | insulin like growth factor binding protein 7 | Extracellular Space | transporter |
| ITIH1_HUMAN | inter-alpha-trypsin inhibitor heavy chain 1 | Extracellular Space | other |
| ITIH2_HUMAN | inter-alpha-trypsin inhibitor heavy chain 2 | Extracellular Space | other |
| ITIH3_HUMAN | inter-alpha-trypsin inhibitor heavy chain 3 | Extracellular Space | other |
| ITIH4_HUMAN | inter-alpha-trypsin inhibitor heavy chain family member 4 | Extracellular Space | other |
| PLAK_HUMAN | junction plakoglobin | Other | other |
| KLKB1_HUMAN | kallikrein B1 | Extracellular Space | peptidase |
| KPRP_HUMAN | keratinocyte proline-rich protein | Cytoplasm | other |
| K2C1_HUMAN | keratin 1, type II | Cytoplasm | other |
| K1C9_HUMAN | keratin 9, type I | Cytoplasm | other |
| LAMA3_HUMAN | laminin subunit alpha 3 | Extracellular Space | other |
| LAMB1_HUMAN | laminin subunit beta 1 | Extracellular Space | other |
| LAMB3_HUMAN | laminin subunit beta 3 | Extracellular Space | transporter |
| LAMC2_HUMAN | laminin subunit gamma 2 | Extracellular Space | other |
| LDHA_HUMAN | lactate dehydrogenase A | Cytoplasm | enzyme |
| LDHB_HUMAN | lactate dehydrogenase B | Cytoplasm | enzyme |
| LRP1_HUMAN | LDL receptor related protein 1 | Plasma Membrane | transmembrane receptor |
| TRFL_HUMAN | lactotransferrin | Extracellular Space | peptidase |
| LUM_HUMAN | lumican | Extracellular Space | other |
| LYSC_HUMAN | lysozyme | Extracellular Space | enzyme |
| MASP1_HUMAN | mannan-binding lectin serine peptidase 1 (C4/C2 activating component of Ra-reactive factor) | Extracellular Space | peptidase |
| MAOX_HUMAN | malic enzyme 1, NADP(+)-dependent, cytosolic | Cytoplasm | enzyme |
| MMP2_HUMAN | matrix metallopeptidase 2 | Extracellular Space | peptidase |
| MRC2_HUMAN | mannose receptor, C type 2 | Plasma Membrane | transmembrane receptor |
| HGFL_HUMAN | macrophage stimulating 1 | Extracellular Space | growth factor |
| NCAM1_HUMAN | neural cell adhesion molecule 1 | Plasma Membrane | other |
| NELL2_HUMAN | neural EGFL like 2 | Extracellular Space | other |
| NEO1_HUMAN | neogenin 1 | Plasma Membrane | transcription regulator |
| MIME_HUMAN | osteoglycin | Extracellular Space | growth factor |
| PARVA_HUMAN | parvin alpha | Cytoplasm | other |
| PCOC1_HUMAN | procollagen C-endopeptidase enhancer | Extracellular Space | other |
| PROF1_HUMAN | profilin 1 | Cytoplasm | other |
| PGK1_HUMAN | phosphoglycerate kinase 1 | Cytoplasm | kinase |
| PGM1_HUMAN | phosphoglucomutase 1 | Cytoplasm | enzyme |
| KPYM_HUMAN | pyruvate kinase, muscle | Cytoplasm | kinase |
| PLMN_HUMAN | plasminogen | Extracellular Space | peptidase |
| PLST_HUMAN | plastin 3 | Cytoplasm | other |
| PLTP_HUMAN | phospholipid transfer protein | Extracellular Space | enzyme |
| POSTN_HUMAN | periostin, osteoblast specific factor | Extracellular Space | other |
| PPIA_HUMAN | peptidylprolyl isomerase A | Cytoplasm | enzyme |
| PROS_HUMAN | protein S (alpha) | Extracellular Space | other |
| PYGB_HUMAN | phosphorylase, glycogen; brain | Cytoplasm | enzyme |
| PZP_HUMAN | pregnancy-zone protein | Extracellular Space | other |
| QSOX1_HUMAN | quiescin sulfhydryl oxidase 1 | Cytoplasm | enzyme |
| RAB8B_HUMAN | RAB8B, member RAS oncogene family | Cytoplasm | enzyme |
| RET4_HUMAN | retinol binding protein 4 | Extracellular Space | transporter |
| RGN_HUMAN | regucalcin | Nucleus | enzyme |
| S10A8_HUMAN | S100 calcium binding protein A8 | Cytoplasm | other |
| ZPI_HUMAN | serpin peptidase inhibitor, clade A (alpha-1 antiproteinase, antitrypsin), member 10 | Extracellular Space | other |
| IPSP_HUMAN | serpin peptidase inhibitor, clade A (alpha-1 antiproteinase, antitrypsin), member 5 | Extracellular Space | other |
| THBG_HUMAN | serpin peptidase inhibitor, clade A (alpha-1 antiproteinase, antitrypsin), member 7 | Extracellular Space | transporter |
| ANT3_HUMAN | serpin peptidase inhibitor, clade C (antithrombin), member 1 | Extracellular Space | enzyme |
| HEP2_HUMAN | serpin peptidase inhibitor, clade D (heparin cofactor), member 1 | Extracellular Space | other |
| PAI1_HUMAN | serpin peptidase inhibitor, clade E (nexin, plasminogen activator inhibitor type 1), member 1 | Extracellular Space | other |
| PEDF_HUMAN | serpin peptidase inhibitor, clade F (alpha-2 antiplasmin, pigment epithelium derived factor), member 1 | Extracellular Space | other |
| A2AP_HUMAN | serpin peptidase inhibitor, clade F (alpha-2 antiplasmin, pigment epithelium derived factor), member 2 | Extracellular Space | other |
| SHBG_HUMAN | sex hormone-binding globulin | Extracellular Space | other |
| SPRR3_HUMAN | small proline-rich protein 3 | Cytoplasm | other |
| ST1E1_HUMAN | sulfotransferase family 1E member 1 | Cytoplasm | enzyme |
| TRFE_HUMAN | transferrin | Extracellular Space | transporter |
| THYG_HUMAN | thyroglobulin | Extracellular Space | other |
| BGH3_HUMAN | transforming growth factor beta induced | Extracellular Space | other |
| TSP1_HUMAN | thrombospondin 1 | Extracellular Space | other |
| TSP4_HUMAN | thrombospondin 4 | Extracellular Space | other |
| TIMP3_HUMAN | TIMP metallopeptidase inhibitor 3 | Extracellular Space | other |
| TLN1_HUMAN | talin 1 | Plasma Membrane | other |
| TITIN_HUMAN | titin | Cytoplasm | kinase |
| TBA1B_HUMAN | tubulin alpha 1b | Cytoplasm | other |
| TBB5_HUMAN | tubulin beta class I | Cytoplasm | other |
| TBB1_HUMAN | tubulin beta 1 class VI | Cytoplasm | other |
| UGDH_HUMAN | UDP-glucose 6-dehydrogenase | Nucleus | enzyme |
| VCAM1_HUMAN | vascular cell adhesion molecule 1 | Plasma Membrane | transmembrane receptor |
| VINC_HUMAN | vinculin | Plasma Membrane | enzyme |
| VNN1_HUMAN | vanin 1 | Plasma Membrane | enzyme |
| VTNC_HUMAN | vitronectin | Extracellular Space | other |
| VWF_HUMAN | von Willebrand factor | Extracellular Space | other |
| 1433E_HUMAN | tyrosine 3-monooxygenase/tryptophan 5-monooxygenase activation protein, epsilon | Cytoplasm | other |
| 1433Z_HUMAN | tyrosine 3-monooxygenase/tryptophan 5-monooxygenase activation protein, zeta | Cytoplasm | enzyme |

| **Treatment** | | | |
| --- | --- | --- | --- |
| **ID** | **Entrez Gene Name** | **Location** | **Type(s)** |
| A2MG_HUMAN | alpha-2-macroglobulin | Extracellular Space | transporter |
| ACES_HUMAN | acetylcholinesterase (Yt blood group) | Plasma Membrane | enzyme |
| ACTB_HUMAN | actin, beta | Cytoplasm | other |
| ACTN1_HUMAN | actinin, alpha 1 | Cytoplasm | transcription regulator |
| ACTN4_HUMAN | actinin, alpha 4 | Cytoplasm | transcription regulator |
| ATS13_HUMAN | ADAM metallopeptidase with thrombospondin type 1 motif 13 | Extracellular Space | peptidase |
| ADHX_HUMAN | alcohol dehydrogenase 5 (class III), chi polypeptide | Cytoplasm | enzyme |
| AFAM_HUMAN | afamin | Extracellular Space | transporter |
| FETA_HUMAN | alpha fetoprotein | Extracellular Space | transporter |
| SAHH_HUMAN | adenosylhomocysteinase | Cytoplasm | enzyme |
| FETUA_HUMAN | alpha-2-HS-glycoprotein | Extracellular Space | other |
| AL9A1_HUMAN | aldehyde dehydrogenase 9 family member A1 | Cytoplasm | enzyme |
| ALDOA_HUMAN | aldolase, fructose-bisphosphate A | Cytoplasm | enzyme |
| AMYP_HUMAN | amylase, alpha 2A (pancreatic) | Extracellular Space | enzyme |
| ANXA1_HUMAN | annexin A1 | Plasma Membrane | enzyme |
| ANXA2_HUMAN | annexin A2 | Plasma Membrane | other |
| AOC3_HUMAN | amine oxidase, copper containing 3 | Plasma Membrane | enzyme |
| APOA1_HUMAN | apolipoprotein A-I | Extracellular Space | transporter |
| APOA4_HUMAN | apolipoprotein A-IV | Extracellular Space | transporter |
| APOB_HUMAN | apolipoprotein B | Extracellular Space | transporter |
| APOE_HUMAN | apolipoprotein E | Extracellular Space | transporter |
| APOH_HUMAN | apolipoprotein H | Extracellular Space | transporter |
| APOM_HUMAN | apolipoprotein M | Plasma Membrane | transporter |
| BHMT1_HUMAN | betaine--homocysteine S-methyltransferase | Cytoplasm | enzyme |
| RIMB1_HUMAN | benzodiazepine receptor (peripheral) associated protein 1 | Cytoplasm | other |
| CR063_HUMAN | chromosome 18 open reading frame 63 | Other | other |
| C1QT3_HUMAN | C1q and tumor necrosis factor related protein 3 | Extracellular Space | other |
| CO2_HUMAN | complement component 2 | Extracellular Space | peptidase |
| CO3_HUMAN | complement component 3 | Extracellular Space | peptidase |
| CO4A_HUMAN | complement component 4B (Chido blood group) | Extracellular Space | other |
| CO5_HUMAN | complement component 5 | Extracellular Space | cytokine |
| CO7_HUMAN | complement component 7 | Extracellular Space | other |
| CO8A_HUMAN | complement component 8, alpha polypeptide | Extracellular Space | other |
| CO8B_HUMAN | complement component 8, beta polypeptide | Extracellular Space | other |
| CO9_HUMAN | complement component 9 | Extracellular Space | other |
| CAB39_HUMAN | calcium binding protein 39 | Cytoplasm | enzyme |
| CAP1_HUMAN | CAP, adenylate cyclase-associated protein 1 (yeast) | Plasma Membrane | other |
| CCD80_HUMAN | coiled-coil domain containing 80 | Nucleus | other |
| CD109_HUMAN | CD109 molecule | Plasma Membrane | other |
| CADH6_HUMAN | cadherin 6 | Plasma Membrane | other |
| CFAB_HUMAN | complement factor B | Extracellular Space | peptidase |
| CFAI_HUMAN | complement factor I | Extracellular Space | peptidase |
| COF1_HUMAN | cofilin 1 (non-muscle) | Nucleus | other |
| CHIA_HUMAN | chitinase, acidic | Extracellular Space | enzyme |
| CLC11_HUMAN | C-type lectin domain family 11 member A | Extracellular Space | growth factor |
| TETN_HUMAN | C-type lectin domain family 3 member B | Extracellular Space | other |
| CLUS_HUMAN | clusterin | Cytoplasm | other |
| CNDP2_HUMAN | CNDP dipeptidase 2 (metallopeptidase M20 family) | Cytoplasm | peptidase |
| CNTN1_HUMAN | contactin 1 | Plasma Membrane | enzyme |
| COAA1_HUMAN | collagen, type X, alpha 1 | Extracellular Space | other |
| CO1A1_HUMAN | collagen, type I, alpha 1 | Extracellular Space | other |
| CO1A2_HUMAN | collagen, type I, alpha 2 | Extracellular Space | other |
| CO2A1_HUMAN | collagen, type II, alpha 1 | Extracellular Space | other |
| CO3A1_HUMAN | collagen, type III, alpha 1 | Extracellular Space | other |
| CO5A1_HUMAN | collagen, type V, alpha 1 | Extracellular Space | other |
| CO6A1_HUMAN | collagen, type VI, alpha 1 | Extracellular Space | other |
| CO6A3_HUMAN | collagen, type VI, alpha 3 | Extracellular Space | other |
| COMP_HUMAN | cartilage oligomeric matrix protein | Extracellular Space | other |
| COR1C_HUMAN | coronin 1C | Cytoplasm | other |
| CERU_HUMAN | ceruloplasmin (ferroxidase) | Extracellular Space | enzyme |
| CBPN_HUMAN | carboxypeptidase N subunit 1 | Extracellular Space | peptidase |
| DPYS_HUMAN | dihydropyrimidinase | Cytoplasm | enzyme |
| EF2_HUMAN | eukaryotic translation elongation factor 2 | Cytoplasm | translation regulator |
| FBLN3_HUMAN | EGF containing fibulin-like extracellular matrix protein 1 | Extracellular Space | enzyme |
| IF4A2_HUMAN | eukaryotic translation initiation factor 4A2 | Cytoplasm | translation regulator |
| ENOA_HUMAN | enolase 1, (alpha) | Cytoplasm | enzyme |
| EZRI_HUMAN | ezrin | Plasma Membrane | other |
| FA10_HUMAN | coagulation factor X | Extracellular Space | peptidase |
| FA11_HUMAN | coagulation factor XI | Extracellular Space | peptidase |
| F13A_HUMAN | coagulation factor XIII, A1 polypeptide | Extracellular Space | enzyme |
| THRB_HUMAN | coagulation factor II, thrombin | Extracellular Space | peptidase |
| FA5_HUMAN | coagulation factor V | Plasma Membrane | enzyme |
| SEPR_HUMAN | fibroblast activation protein alpha | Cytoplasm | peptidase |
| FBLN1_HUMAN | fibulin 1 | Extracellular Space | other |
| FBN1_HUMAN | fibrillin 1 | Extracellular Space | other |
| FIBB_HUMAN | fibrinogen beta chain | Extracellular Space | other |
| FIBG_HUMAN | fibrinogen gamma chain | Extracellular Space | other |
| FILA_HUMAN | filaggrin | Cytoplasm | other |
| FILA2_HUMAN | filaggrin family member 2 | Cytoplasm | other |
| FLNA_HUMAN | filamin A | Cytoplasm | other |
| FINC_HUMAN | fibronectin 1 | Extracellular Space | enzyme |
| G3P_HUMAN | glyceraldehyde-3-phosphate dehydrogenase | Cytoplasm | enzyme |
| VTDB_HUMAN | group-specific component (vitamin D binding protein) | Extracellular Space | transporter |
| GDIB_HUMAN | GDP dissociation inhibitor 2 | Cytoplasm | other |
| GPX3_HUMAN | glutathione peroxidase 3 | Extracellular Space | enzyme |
| GELS_HUMAN | gelsolin | Extracellular Space | other |
| GSTM5_HUMAN | glutathione S-transferase mu 5 | Cytoplasm | enzyme |
| HBD_HUMAN | hemoglobin subunit delta | Other | transporter |
| HGFA_HUMAN | HGF activator | Extracellular Space | peptidase |
| HS90B_HUMAN | heat shock protein 90kDa alpha family class B member 1 | Cytoplasm | enzyme |
| HSP7C_HUMAN | heat shock protein family A (Hsp70) member 8 | Cytoplasm | enzyme |
| IGF2_HUMAN | insulin like growth factor 2 | Extracellular Space | growth factor |
| MPRI_HUMAN | insulin like growth factor 2 receptor | Plasma Membrane | transmembrane receptor |
| IBP2_HUMAN | insulin like growth factor binding protein 2 | Extracellular Space | other |
| ITA2_HUMAN | integrin subunit alpha 2 | Plasma Membrane | transmembrane receptor |
| ITIH1_HUMAN | inter-alpha-trypsin inhibitor heavy chain 1 | Extracellular Space | other |
| ITIH2_HUMAN | inter-alpha-trypsin inhibitor heavy chain 2 | Extracellular Space | other |
| ITIH3_HUMAN | inter-alpha-trypsin inhibitor heavy chain 3 | Extracellular Space | other |
| ITIH4_HUMAN | inter-alpha-trypsin inhibitor heavy chain family member 4 | Extracellular Space | other |
| PLAK_HUMAN | junction plakoglobin | Other | other |
| KLKB1_HUMAN | kallikrein B1 | Extracellular Space | peptidase |
| K2C1_HUMAN | keratin 1, type II | Cytoplasm | other |
| K1C9_HUMAN | keratin 9, type I | Cytoplasm | other |
| LAMA3_HUMAN | laminin subunit alpha 3 | Extracellular Space | other |
| LAMB1_HUMAN | laminin subunit beta 1 | Extracellular Space | other |
| LAMB3_HUMAN | laminin subunit beta 3 | Extracellular Space | transporter |
| LAMC1_HUMAN | laminin subunit gamma 1 | Extracellular Space | other |
| LAMC2_HUMAN | laminin subunit gamma 2 | Extracellular Space | other |
| LDHA_HUMAN | lactate dehydrogenase A | Cytoplasm | enzyme |
| LDHB_HUMAN | lactate dehydrogenase B | Cytoplasm | enzyme |
| LRP1_HUMAN | LDL receptor related protein 1 | Plasma Membrane | transmembrane receptor |
| TRFL_HUMAN | lactotransferrin | Extracellular Space | peptidase |
| LUM_HUMAN | lumican | Extracellular Space | other |
| MASP1_HUMAN | mannan-binding lectin serine peptidase 1 (C4/C2 activating component of Ra-reactive factor) | Extracellular Space | peptidase |
| MMP2_HUMAN | matrix metallopeptidase 2 | Extracellular Space | peptidase |
| MRC2_HUMAN | mannose receptor, C type 2 | Plasma Membrane | transmembrane receptor |
| HGFL_HUMAN | macrophage stimulating 1 | Extracellular Space | growth factor |
| MYH2_HUMAN | myosin, heavy chain 2, skeletal muscle, adult | Cytoplasm | enzyme |
| NEO1_HUMAN | neogenin 1 | Plasma Membrane | transcription regulator |
| NDKB_HUMAN | NME/NM23 nucleoside diphosphate kinase 2 | Nucleus | kinase |
| MIME_HUMAN | osteoglycin | Extracellular Space | growth factor |
| PCLO_HUMAN | piccolo presynaptic cytomatrix protein | Cytoplasm | transporter |
| PCOC1_HUMAN | procollagen C-endopeptidase enhancer | Extracellular Space | other |
| PFKAL_HUMAN | phosphofructokinase, liver | Cytoplasm | kinase |
| PGK1_HUMAN | phosphoglycerate kinase 1 | Cytoplasm | kinase |
| PGM1_HUMAN | phosphoglucomutase 1 | Cytoplasm | enzyme |
| KPYM_HUMAN | pyruvate kinase, muscle | Cytoplasm | kinase |
| PAFA_HUMAN | phospholipase A2 group VII | Extracellular Space | enzyme |
| PLMN_HUMAN | plasminogen | Extracellular Space | peptidase |
| PLST_HUMAN | plastin 3 | Cytoplasm | other |
| PLTP_HUMAN | phospholipid transfer protein | Extracellular Space | enzyme |
| PLVAP_HUMAN | plasmalemma vesicle associated protein | Plasma Membrane | other |
| POSTN_HUMAN | periostin, osteoblast specific factor | Extracellular Space | other |
| PPIA_HUMAN | peptidylprolyl isomerase A | Cytoplasm | enzyme |
| PPIB_HUMAN | peptidylprolyl isomerase B | Cytoplasm | enzyme |
| PRDX1_HUMAN | peroxiredoxin 1 | Cytoplasm | enzyme |
| PRDX2_HUMAN | peroxiredoxin 2 | Cytoplasm | enzyme |
| PSA1_HUMAN | proteasome subunit alpha 1 | Cytoplasm | peptidase |
| PSA4_HUMAN | proteasome subunit alpha 4 | Cytoplasm | peptidase |
| PSA6_HUMAN | proteasome subunit alpha 6 | Cytoplasm | peptidase |
| PSA7_HUMAN | proteasome subunit alpha 7 | Cytoplasm | peptidase |
| PSMD5_HUMAN | proteasome 26S subunit, non-ATPase 5 | Other | other |
| PYGL_HUMAN | phosphorylase, glycogen, liver | Cytoplasm | enzyme |
| PZP_HUMAN | pregnancy-zone protein | Extracellular Space | other |
| QSOX1_HUMAN | quiescin sulfhydryl oxidase 1 | Cytoplasm | enzyme |
| RAB8B_HUMAN | RAB8B, member RAS oncogene family | Cytoplasm | enzyme |
| RAN_HUMAN | RAN, member RAS oncogene family | Nucleus | enzyme |
| RAP1B_HUMAN | RAP1B, member of RAS oncogene family | Cytoplasm | enzyme |
| RET4_HUMAN | retinol binding protein 4 | Extracellular Space | transporter |
| RGN_HUMAN | regucalcin | Nucleus | enzyme |
| S10A7_HUMAN | S100 calcium binding protein A7 | Cytoplasm | other |
| S10A9_HUMAN | S100 calcium binding protein A9 | Cytoplasm | other |
| ZPI_HUMAN | serpin peptidase inhibitor, clade A (alpha-1 antiproteinase, antitrypsin), member 10 | Extracellular Space | other |
| IPSP_HUMAN | serpin peptidase inhibitor, clade A (alpha-1 antiproteinase, antitrypsin), member 5 | Extracellular Space | other |
| THBG_HUMAN | serpin peptidase inhibitor, clade A (alpha-1 antiproteinase, antitrypsin), member 7 | Extracellular Space | transporter |
| ANT3_HUMAN | serpin peptidase inhibitor, clade C (antithrombin), member 1 | Extracellular Space | enzyme |
| PAI1_HUMAN | serpin peptidase inhibitor, clade E (nexin, plasminogen activator inhibitor type 1), member 1 | Extracellular Space | other |
| PEDF_HUMAN | serpin peptidase inhibitor, clade F (alpha-2 antiplasmin, pigment epithelium derived factor), member 1 | Extracellular Space | other |
| A2AP_HUMAN | serpin peptidase inhibitor, clade F (alpha-2 antiplasmin, pigment epithelium derived factor), member 2 | Extracellular Space | other |
| TAGL2_HUMAN | transgelin 2 | Cytoplasm | other |
| TRFE_HUMAN | transferrin | Extracellular Space | transporter |
| TFR1_HUMAN | transferrin receptor | Plasma Membrane | transporter |
| THYG_HUMAN | thyroglobulin | Extracellular Space | other |
| BGH3_HUMAN | transforming growth factor beta induced | Extracellular Space | other |
| TSP1_HUMAN | thrombospondin 1 | Extracellular Space | other |
| TSP4_HUMAN | thrombospondin 4 | Extracellular Space | other |
| TIMP3_HUMAN | TIMP metallopeptidase inhibitor 3 | Extracellular Space | other |
| TLN1_HUMAN | talin 1 | Plasma Membrane | other |
| TENX_HUMAN | tenascin XB | Extracellular Space | other |
| TPM1_HUMAN | tropomyosin 1 (alpha) | Cytoplasm | other |
| TITIN_HUMAN | titin | Cytoplasm | kinase |
| TBA1B_HUMAN | tubulin alpha 1b | Cytoplasm | other |
| TBA4A_HUMAN | tubulin alpha 4a | Cytoplasm | other |
| TBB5_HUMAN | tubulin beta class I | Cytoplasm | other |
| TBB1_HUMAN | tubulin beta 1 class VI | Cytoplasm | other |
| RL40_HUMAN | ubiquitin A-52 residue ribosomal protein fusion product 1 | Cytoplasm | enzyme |
| UGDH_HUMAN | UDP-glucose 6-dehydrogenase | Nucleus | enzyme |
| VINC_HUMAN | vinculin | Plasma Membrane | enzyme |
| VTNC_HUMAN | vitronectin | Extracellular Space | other |
| VWF_HUMAN | von Willebrand factor | Extracellular Space | other |
| WDR1_HUMAN | WD repeat domain 1 | Extracellular Space | other |
| 1433E_HUMAN | tyrosine 3-monooxygenase/tryptophan 5-monooxygenase activation protein, epsilon | Cytoplasm | other |
| 1433T_HUMAN | tyrosine 3-monooxygenase/tryptophan 5-monooxygenase activation protein, theta | Cytoplasm | other |
| 1433Z_HUMAN | tyrosine 3-monooxygenase/tryptophan 5-monooxygenase activation protein, zeta | Cytoplasm | enzyme |
